# Clinical and laboratory predictors of death in African children with features of severe malaria: a systematic review and meta-analysis

**DOI:** 10.1186/s12916-017-0906-5

**Published:** 2017-08-03

**Authors:** Paulina Sypniewska, Jose F. Duda, Isabella Locatelli, Clotilde Rambaud Althaus, Fabrice Althaus, Blaise Genton

**Affiliations:** 1 Department of Ambulatory Care and Community Medicine, University of Lausanne, Lausanne, Switzerland; 2 RWTH Aachen University, Aachen, Germany; 3 International Committee of the Red Cross, Geneva, Switzerland; 4 Swiss Tropical and Public Health Institute, University of Basel, Basel, Switzerland; 5 Division of Infectious Diseases, University Hospital, Lausanne, Switzerland

**Keywords:** Severe malaria, Predictors, Death, Mortality, Systematic review

## Abstract

**Background:**

The criteria for defining severe malaria have evolved over the last 20 years. We aimed to assess the strength of association of death with features currently characterizing severe malaria through a systematic review and meta-analysis.

**Method:**

Electronic databases (Medline, Embase, Cochrane Database of Systematic Reviews, Thomson Reuters Web of Knowledge) were searched to identify publications including African children with severe malaria. PRISMA guidelines were followed. Selection was based on design (epidemiological, clinical and treatment studies), setting (Africa), participants (children < 15 years old with severe malaria), outcome (survival/death rate), and prognostic indicators (clinical and laboratory features). Quality assessment was performed following the criteria of the 2011 Quality Assessment of Diagnostic Accuracy Studies (QUADAS-2). Odds ratios (ORs) were calculated for each study and prognostic indicator, and, when a test was assessed in at least two studies, pooled estimates of ORs were computed using fixed- or random-effects meta-analysis.

**Results:**

A total of 601 articles were identified and screened and 30 publications were retained. Features with the highest pooled ORs were renal failure (5.96, 95% CI 2.93–12.11), coma score (4.83, 95% CI 3.11–7.5), hypoglycemia (4.59, 95% CI 2.68–7.89), shock (4.31, 95% CI 2.15–8.64), and deep breathing (3.8, 95% CI 3.29–4.39). Only half of the criteria had an OR > 2. Features with the lowest pooled ORs were impaired consciousness (0.58, 95% CI 0.25–1.37), severe anemia (0.76, 95% CI 0.5– 1.13), and prostration (1.12, 95% CI 0.45–2.82).

**Conclusion:**

The findings of this meta-analysis show that the strength of association between the criteria defining severe malaria and death is quite variable for each clinical and/or laboratory feature (OR ranging from 0.58 to 5.96). This ranking allowed the identification of features weakly associated with death, such as impaired consciousness and prostration, which could assist to improve case definition, and thus optimize antimalarial treatment.

**Electronic supplementary material:**

The online version of this article (doi:10.1186/s12916-017-0906-5) contains supplementary material, which is available to authorized users.

## Background

Severe malaria accounted for approximately 2 million out of 207 million estimated malaria cases in 2012 [1]. In areas with intense and stable transmission, children under the age of 5 years carry the heaviest burden, especially in the sub-Saharan region [2]. Although a correct and prompt diagnosis of severe malaria is crucial for prescribing appropriate therapy, and thus for reducing mortality, the parenteral administration of first-line treatment often remains a challenge in resource poor settings. Improved targeting of children who would benefit most from parenteral treatment rather than oral treatment would help the overall management of malaria cases.

A child is diagnosed with severe malaria when asexual *P. falciparum* parasitemia is detected in the peripheral blood smear or confirmed by a rapid diagnostic test, there is no other cause for its symptoms, and at least one of impaired consciousness, respiratory distress, multiple convulsions, prostration, shock, pulmonary edema, abnormal bleeding, jaundice, severe anemia, hypoglycemia, acidosis, hyperlactatemia, renal impairment, or hyperparasitemia is present. These criteria reflect the definition of severe malaria established by the World Health Organization (WHO) in 2000, according to which any child with positive blood parasitemia and at least one of abovementioned criteria is qualified to receive parenteral treatment [3].

In recent years, a decrease in the case fatality rate of malaria has been observed [4]. The reasons for this improvement are not entirely clear, but introduction of drugs with increased efficacy [5, 6] and effective control programs [7] have certainly played a crucial role. A reduction in the case fatality rate of severe malaria has also been documented in controlled trials [5, 6]. A potential confounder for this observed reduction may be related to a selection bias due to a shift in severe malaria case definition. In 1990, the WHO set the criteria for a strict definition of severe malaria for research and epidemiological purposes [8]. In 2000, new neurological criteria, i.e., prostration and impaired consciousness, were introduced into the definition [9], and recent works have relied on a wider pragmatic case definition. For example, the Severe Malaria in African Children (SMAC) studies included children with *P. falciparum* detected on blood smear and classified as “being severely ill enough to be hospitalized”, without further specifications [10].

In this context, we conducted a systematic review and meta-analysis to better understand the prognostic value of clinical and laboratory findings used to diagnose severe malaria in African children. This assessment was aimed at refining the commonly employed definition of severe malaria to then explore the possibility to define ‘moderately severe malaria’ cases that could benefit from much more accessible oral treatment.

## Methods

### Search strategy and sources

We performed a systematic literature search using Medline, Embase, Cochrane Database of Systematic Reviews, and Thomson Reuters Web of Knowledge. Study selection followed the Preferred Reporting Items for Systemic Reviews and Meta-Analyses (PRISMA) guidelines [11]. The first search was undertaken in January 2014, with an update in February 2015. We searched Medline and Embase using Medical Subject Headings and subheadings used for indexing articles. We combined the following terms: “malaria/complications OR malaria/mortality” AND “treatment outcome” AND “infant, newborn OR infant OR child OR adolescent”. In the Cochrane Database, we looked for the words “malaria and children” in the main title of the review. We searched the Thomson Reuters Web of Knowledge using the words “malaria child”, “Africa”, “mortality” and “complications”. We did not put any language or time restrictions on the search and we expanded it by examining the reference list of the selected studies. Additionally, we used three landmark articles [10, 12, 13] on severe malaria in African children to search for citations closely related to the selected article using the PubMed option “Related citations”.

### Inclusion and exclusion criteria

Studies reporting clinical and laboratory variables, including at least 100 children aged < 15 years who were diagnosed with severe malaria according to the WHO definitions, and which allowed reconstructing of two-by-two tables made up of outcome (survival/death) and presence/absence of prognostic indicator, were included in this review. Controlled trials, non-controlled trials, cohort studies, case control studies and case series, both prospective and retrospective, were considered. When necessary, authors were contacted to obtain data to construct two-by-two tables. Two independent reviewers (BG and JD) conducted this search. Two [5, 10] of the included studies served as reference publications for other enclosed publications, although no direct prognostic indicators could be extracted. Three selected studies [13–15] considered either partial or the whole population included originally in the study comparing artesunate with quinine in severe malaria treatment in Africa (known as the AQUAMAT study). In this case, the study with a greater number of study subjects with available clinical or laboratory features associated with death was selected. Two articles [16, 17] encompassed the same study population though they focused on distinct clinical or laboratory variables; thus, both of them were retained. In addition, 356 out of 2901 children enrolled in a study in The Gambia [18] also participated in the AQUAMAT study, which leads to duplication of the subjects included in these two large studies.

Finally, in view of the size of the comprised population, we also considered data from the SMAC studies [19] in our systematic review, although study inclusion criteria did not fully comply with the strict WHO definition of severe malaria. Therefore, we performed separate analyses with and without the SMAC studies.

### Quality assessment

Quality of selected studies and their risk of biases were assessed by applying the 2011 revised version of the Quality Assessment of Diagnostic Accuracy Studies (QUADAS-2) tool [20], which was adjusted to the particularity of this review following the recommendation of the Cochrane Collaboration (details in Additional file 1) [21]. When the patients’ inclusion criteria differed from the WHO criteria, we reported in the methodological quality assessment that there were great concerns about the applicability of the results to the research question. Regarding prognostic indicators, clinical and laboratory features were assessed separately. Furthermore, any reported death was considered as a reference standard. Studies including less than 80% of enrolled patients were labeled as highly biased. Quality assessment was performed by one reviewer (PS) and checked by a second reviewer (BG). Any disagreements were resolved through discussion and consensus.

### Data extraction

Data on clinical features among children who survived or died were extracted by one reviewer (PS) using a standardized data extraction form and checked by the second (JD), as well as on random basis by the third (BG) reviewer. Information on characteristics (design, year of publication, study country, healthcare setting), study population (size, age range, mortality, inclusion and exclusion criteria), and prognostic indicators was gathered. Any identified errors were re-examined and corrected accordingly.

### Statistical analysis

A two-by-two table including crossing variables, *index test* (0,1) and *death* (0,1), was constructed for each prognostic indicator. Odds ratios (ORs) were calculated to measure the association of each prognostic indicator with death. When a prognostic indicator was assessed in at least two studies, pooled estimates of ORs were calculated. A random effects meta-analysis was performed in the case of a significant heterogeneity among studies (*P* < 0.05). Otherwise, the fixed-effect approach was preferred. *Metan* command in STATA version 12 was used to perform these meta-analyses [22]. Results for all predictors were summarized in a Forrest plot, ordering markers from the least to the most strongly associated with death. The size of each predictor’s box is proportional to the global sample size of studies involved in the corresponding summary ORs. Two separate analyses were conducted; one enclosing additional findings derived from the SMAC studies and one without it, covering studies that referred strictly to the definition of WHO as diagnosis criteria. Prognostic indicators with definitive thresholds and few without single definition (acidosis, hyperparasitemia, renal failure, respiratory distress, shock) were pooled for the usage of this analysis. The combination of symptoms was not analyzed in this systematic review due to unavailability of individual records.

## Results

**Fig. 1:**
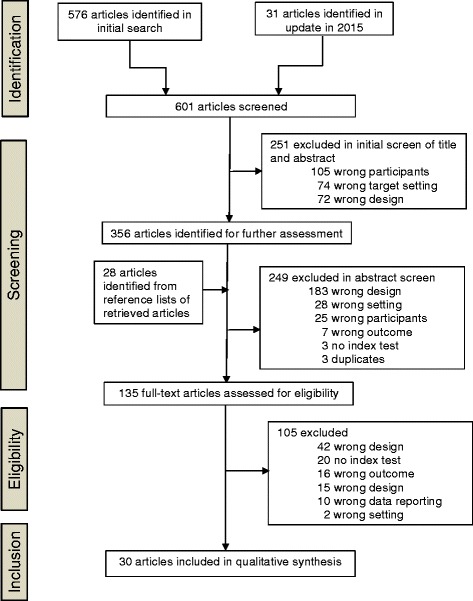
Flow diagram of the study selection process. Only the first reason for exclusion (as ordered in Appendix) is reported

A total of 601 studies were identified and screened in the systematic database search. Through the selection process presented in the flow diagram (Fig. 1), 30 titles [5, 10, 13–19, 23–43] published between 1994 and 2014 were selected and used to identify predictors; 28 were finally included in the meta-analysis (no direct data could be extracted from two referral studies). Overall, 90% of eligible studies were reported in English and 10% in French. The characteristics of the studies are outlined in Table 1. The summary of quality assessment of analyzed studies, according to the QUADAS-2 tool, is presented in Table 2. The detailed analysis of each study according to the QUADAS-2 tool was captured in Additional file 2.

**Table 1:** Characteristics of included studies

Reference	Design	Setting	Age range	Patients (N)	Case fatality rate (in the study)	Inclusion criteria	Exclusion criteria	List of prognostic indicators
Krishna 1994 [23]	Prosp	The Gambia; IPD; Research institute	1.5–12 years	115	18.3%	*P. falciparum*-positive blood film and one or more of the following features: BCS ≤2, parasitemia >100000/μL with 15%, or shock	Other causes of fever or altered consciousness (excluded by examination of blood culture and cerebrospinal fluid)	Included: hyperlactatemia, hypoglycemia, coma (BCS ≤2) Excluded: parasitemia, TNF, IL-1α
English 1996 [16]	Prosp	Kenya; pediatric ward	mean age:31 months	350	8.6%	*P. falciparum* and one or more of the following clinical symptoms: coma, prostration, hyperparasitaemia, respiratory distress	NR	Included: respiratory distress, deep breathing, nasal flaring, acidosis, chest indrawing
English 1997 [17]	Prosp	Kenya; pediatric ward	mean age 31 months	306	8.0%	*P. falciparum* and one or more of the following clinical syndromes: coma, prostration, hyperparasitaemia, respiratory distress	Death prior to admission assessment	Included: coma score Excluded: respiratory distress, acidosis, hyperparasitemia and prostration, respiratory distress and cerebral malaria
Assimadi 1998 [24]	Prosp	Togo; pediatric ward	0–15 years	549	18.9%	WHO (1995) criteria of severe malaria	NR	Included: renal failure, circulatory collapse, abnormal hemorrhage, jaundice, choluria, prostration, impaired consciousness, respiratory distress, hypoglycemia, convulsions, coma score, severe anemia, acidosis
Modiano 1998 [25]	Prosp	Burkina Faso; pediatric ward	6 months to 15 years	800	13.8%	WHO definition of severe malaria	Other detectable infections or causes	Included: prostration, coma, convulsions, anemia, hypoglycemia, pulmonary edema/respiratory distress, spontaneous bleeding, renal failure
Varandas 2000 [26]	RCT	Mozambique; pediatric ward	6–72 months	559	3.6%	Criteria for cerebral malaria: coma without a directional response to a painful stimulus 6 h after the last convulsion, clear CSF, parasitemia or positive PCR to *P. falciparum*; other forms of severe malaria: WHO criteria (1990) and confirmed by parasitemia	Children with a history of measles or measles vaccination in the 4 weeks preceding admission, clinical signs of vitamin A deficiency, signs of kwashiorkor or marasmus or other severe diseases	Included: age, respiratory distress on admission, acidotic breathing on admission, inability to localize painful stimulus on admission, convulsions before admission Excluded: maternal education, poor housing, loss of consciousness before admission, convulsions on admission, not-transfused children
Gérardin 2002 [27]	Prosp	Senegal; pediatric ward	0–15 years	215	12.1%	2000 WHO definition of severe malaria	NR	Included: thrombocytopenia Excluded (no differentiation between severe malaria and malaria): light cerebral disorder, cerebral malaria, convulsions, respiratory distress, severe anemia, jaundice, acidosis, hyperparasitemia, hemoglobinuria, renal failure (abnormal bleeding/collapse/pulmonary edema)
Imbert 2003 [28]	Prosp	Senegal; pediatric ward	<15 years	311	9.0%	*P. falciparum* trophozoites, WHO criteria of severe malaria	Simple malaria cases	Included: impaired consciousness, coma, respiratory distress, convulsions, jaundice, severe anemia, hyperparasitemia, hypoglycemia, prostration, hemoglobinuria, renal failure, shock, abnormal hemorrhage, pulmonary edema, pupillary anomalies, thrombocytopenia, leukocytosis, co-infection, hyperpyrexia Excluded: acidosis
Maitland 2003 [29]	Retro	Kenya; pediatric high-dependency unit	75% <36 months	515	12.8%	*P. falciparum,* and one or more of the following: prostration, coma, prolonged or recurrent seizures, respiratory distress, circulatory collapse, anemia, jaundice	NR	Included: impaired consciousness, acidosis, severe anemia, jaundice, hypoglycemia, sex, age, wasting, shock, deep breathing, convulsions Excluded: hypoglycemia
Mockenhaupt 2004 [30]	Prosp	Ghana; pediatric ward	6–102 months	285	11.2%	asexual *P. falciparum* parasitemia, and one or more of the following WHO (2000) criteria: severe anemia, prostration, respiratory distress, multiple convulsions, impaired consciousness, hemoglobinuria, clinical jaundice, circulatory collapse, abnormal bleeding, pulmonary edema	NR	Included: severe anemia, prostration, respiratory distress, convulsions, impaired consciousness, jaundice, circulatory collapse, hemoglobinuria, coma (BCS ≤2), hyperparasitemia, hypoglycemia, hyperlactatemia, hyperpyrexia
Dzeing-Ella 2005 [31]	Prosp	Gabon; tertiary referral center	0–10 years	576	8.9%	Age 0–10 years, more than two asexual forms of *P. falciparum* on blood film and one or more of the following features: BCS ≤2, convulsions, hyperlactatemia, hypoglycemia, severe anemia	Alternative diagnosis made clinically or by investigation (e.g., CSF examination, chest radiography, blood culture)	Included: coma, respiratory distress, severe anemia, hypoglycemia, hyperlactatemia, convulsions, sex
Gay-Andrieu 2005 [32]	Prosp	Niger; pediatric ward	3–60 months	114	21.0%	*P. falciparum* and at least one of the following clinical or biological criteria: coma (BCS ≤2), impaired consciousness (BCS >2 and <5), repeated convulsions, prostration, respiratory distress, jaundice, metabolic acidosis, severe anemia, hyperparasitemia, microscopic haemoglobinuria, renal failure, collapse, abnormal bleeding or pulmonary edema	NR	Included: coma, impaired consciousness, convulsions, respiratory distress, severe anemia, hypoglycemia, hyperparasitemia
Maitland 2005 [33]	RCT	Kenya; pediatric high-dependency unit	median age 2.8 years	150	0.1%	Clinical feature of severe malaria (i.e., prostration, coma, or respiratory distress), and *P. falciparum* parasitemia and metabolic acidosis (base deficit >8 mmol/L) and Hb >50 g/L	Pulmonary edema, edematous malnutrition, papilledema	Included: acidosis
Zeidan 2005 [34]	Prosp, cx	Sudan; IPD	<15 years	543	2.6%	Identification of *P. falciparum* in blood film and presence of any of combined complications of change of behaviors, confusion or drowsiness, altered consciousness or coma, convulsions, hypoglycemia, acidosis, difficulty in breathing, pulmonary edema, oliguria, acute renal failure, severe anemia (hematocrit <20%, Hb <6 g/dL), haemoglobinuria, jaundice, tendency to bleed, and generalized weakness rendering the patient unable to walk or sit up without assistance	NR	Included: prostration, age, hyperpyrexia, convulsions, leukocytosis, coma Excluded: delays, jaundice, anemia, hepatomegaly, splenomegaly, hypoglycemia, hemoglobinuria, fever, vomiting
Bronzan 2007 [35]	Retro	Malawi; pediatric research ward,	≥6 months	1388	16.0%	Presenting one of three severe malaria syndromes: CM, SMA, or CM with SMA and confirmed by *P. falciparum* blood film test	Other identifiable causes of coma (such as hypoglycemia, postictal state, and meningitis)	Included: severe anemia, cerebral malaria (BCS ≤2 persisting for >2 hours after other identifiable causes of coma have been excluded), HIV infection
Issifou 2007 [36]	Prosp	Gabon; medical research unit; IPD	1–120 months	2235	3.0%	Age 1–120 months, “*non per os*” falciparum malaria (patients hospitalized for malaria and treated with intravenous quinine in a 10% glucose infusion)	NR	Included: coma (BCS ≤2), hypoglycemia, severe anemia, respiratory distress
Oduro 2007 [37]	Prosp	Ghana; IPD	6–59 months	868	3.5%	1990 and 2000 WHO severe malaria criteria	NR	Included: coma (BCS <3), severe anemia, respiratory distress, hyperlactatemia, hypoglycemia
Orimadegun 2007 [38]	Retro	Nigeria; tertiary hospital	6 months to 15 years	1806	6.9%	2000 WHO severe malaria criteria	NR	Included: severe anemia, coma (BCS <3), sex Excluded: hypoglycemia, respiratory distress
Bassat 2008 [39]	Retro	Mozambique; district hospital	<15 years	1100	4.4%	Malaria case with at least one of the following criteria: PCV <15%, deep coma (BCS ≤2), prostration, hypoglycemia, convulsions, respiratory distress	Children with malaria parasitaemia for whom the cause of death was not malaria were not considered severe malaria cases	Included: severe anemia, coma, convulsions, hypoglycemia, prostration, respiratory distress, impaired consciousness, jaundice, dehydration
Ranque 2008 [40]	Prosp	Mali; pediatric ward	<15 years	455	16.0%	Fever >38 °C and *P. falciparum* trophozoites, and no suggestion of other diagnosis; all children diagnosed with CM and/or SMA	NR	Included: fever >4 days, personal history of CM, CM history in siblings, coma score, severe anemia, hypoglycemia, dehydration, spleen enlargement, liver enlargement, prior antimalarial treatment, respiratory distress, age group, sex
Ogetii 2010 [41]	Retro	Kenya; pediatric high-dependency unit	0–12 years	1236	10.5%	*P. falciparum* parasitemia plus impaired consciousness and/or respiratory distress	Children admitted to the general ward with malaria parasitaemia who deteriorated after admission fulfilling the definition of severe malaria	Included: hypoglycemia
Camara 2011 [42]	Prosp	Senegal; pediatric ward	0–15 years	162	11.1%	Aged 0–15 years, *P. falciparum*-positive thick drop examination and at least one of the WHO 2000 malaria severity criteria	All children with anti-malarial treatment started less than 72 hours prior to hospitalization	Included: sex, age, impaired consciousness, prostration, respiratory distress, coma, shock, convulsions, jaundice, hypoglycemia, thrombocytopenia, severe anemia, hemoglobinuria
Hendrikson 2012 [15]^a^	RCT	Mozambique; tertiary referral hospital	<15 years	655	10.9%	Children (<15 years) with suspected severe malaria according to modified WHO clinical criteria, positive pLDH-based RDT	If treated parenterally for >24 hours before admission	Included: HIV infection
Hendriksen 2012 [14]^a^	RCT	Mozambique, Gambia, Kenya, Tanzania, Uganda, Rwanda, DRC; pediatric wards	1 month to 15 years	3826	9.9%	Positive *P. falciparum* histidine-rich protein two-based rapid test (Optimal), and at least one of coma, prostration, convulsions, compensated shock, decompensated shock, severe respiratory distress, hypoglycemia, severe symptomatic anemia, blackwater fever, clinical jaundice, hyperparasitemia	If treated parenterally for >24 hours before admission	Included: acidosis, severe anemia, hypoglycemia, hyperparasitemia Excluded: respiratory distress, coma, convulsions, jaundice, shock, black water fever
Jallow 2012 [18]	Prosp obser	Gambia; pediatric ward	4 months to 14 years	2901	13.0%	Asexual *P. falciparum* parasitemia*,* and one or more of the following WHO criteria for severe malaria: severe anemia, respiratory distress, hypoglycemia, decompensated shock, repeated convulsion, acidosis, hyperlactatemia	NR	Included: acidosis, coma score, convulsions, severe anemia, hyperalactatemia, hyperparsitemia, hyperpyrexia, hypoglycemia, impaired consciousness, deep breathing, jaundice, liver enlargement, prostration, renal failure, respiratory distress, shock, spleen enlargement
von Seidlein 2012 [13]^a^	RCT	Gambia, Mozambique, Nigeria, Rwanda, Kenya, DRC, Tanzania, Ghana, Uganda; pediatric wards	<15 years	5426	9.7%	Positive *P. falciparum* histidine-rich protein two-based rapid test (Optimal), and at least one of coma, prostration, convulsions, compensated shock, decompensated shock, severe respiratory distress, hypoglycemia, severe symptomatic anemia, blackwater fever, clinical jaundice, hyperparasitemia	If treated parenterally for >24 hours before admission	Included: convulsions, prostration, coma shock, respiratory distress, deep breathing, jaundice, chronic disease, sex, black water fever Excluded: blood urea nitrogen, base excess, pH, respiratory rate, parasite density, hemoglobin, glucose level, temperature, age, heart rate
Kendjo 2013 [19]^b^	Prosp, cx	Sub-Saharan Africa; hospital research centres	<15 years	26 296	4.3%	Severe *P. falciparum* malaria	NR	Included: seizures prior to admission, vomiting prior to admission, deep breathing, indrawing, irregular breathing, prostration, coma, hyperparasitemia, severe anemia, hypoglycemia Excluded: hyperlactatemia
Orimadegun 2014 [43]	Prosp, cx	Nigeria; tertiary hospital	<5 years	369	8.1%	*P. falciparum* malaria parasitaemia confirmed with blood film microscopy and the presence of any of the defined life-threatening features for malaria according to WHO (2000)	Children who had clinical signs suggestive of cardiac defect and those whose parents refused consent	Included: severe anemia, hyperparasitemia, acidosis, hemoglobinuria, hypoglycemia, coma score, renal impairment, hypoxia Excluded: hypokalemia, hyponatremia, pneumonia, azotemia, wasting

^a^Referral study: Dondorp 2010 [5]

^b^Referral study: Helbok 2009 [10]

Excluded prognostic indicators: not enough data to construct two-by-two tables

*BCS* Blantyre coma scale, *CM* cerebral malaria, *CSF* cerebrospinal fluid, *Cx* cross-sectional, *IPD* In-patient department, *NR* not reported, *Obs* observational, *pLDH* parasite lactate dehydrogenase, *Prosp* prospective, *Retro* retrospective, *RCT* randomized clinical trial, *RDT* rapid diagnostic test, *SMA* severe malarial anemia, *WHO* World Health Organization

**Table 2:** Quality assessment according to the QUADAS-2 tool: potential bias and applicability concerns of included studies (without referral studies)

	Risk of bias					Applicability concerns			
	Patient selection	Clinical predictors of mortality	Laboratory predictors of mortality	Reference standard	Flow and timing	Patient selection	Clinical predictors of mortality	Laboratory predictor of mortality	Reference standard
Krishna et al. (1994) [23]	Low	Low	Low	Low	Low	High	Low	Low	Low
English et al. (1996) [16]	Low	Low	Low	Low	Low	Low	Low	Low	Low
English et al. (1997) [17]	High	Low	NA	Low	Low	Unclear	High	NA	Low
Assimadi et al. (1998) [24]	Low	High	High	Low	Low	Low	High	High	Low
Modiano et al. (1998) [25]	Low	Unclear	Low	Low	Low	Low	Unclear	Low	Low
Varandas et al. (2000) [26]	High	Low	NA	Low	Low	Low	Low	NA	Low
Gérardin et al. (2002) [27]	Low	Low	Low	Low	Low	Low	Low	Low	Low
Imbert et al. (2003) [28]	Low	Low	Low	Low	Low	Low	Low	Low	Low
Maitland et al. (2003) [29]	Low	Low	Low	Low	Low	Low	Low	Unclear	Low
Mockenhaupt et al. (2004) [30]	Low	Low	Low	Low	Low	Low	Low	Low	Low
Dzeing-Ella et al. (2005) [31]	Low	Low	Low	Low	Low	Low	Low	Low	Low
Gay-Andrieu et al. (2005) [32]	High	Unclear	Low	Low	Low	High	Unclear	Low	Low
Maitland et al. (2005) [33]	High	NA	Low	Low	Low	High	NA	Low	Low
Zeidan et al. (2005) [34]	Low	Low	Low	Low	Low	Low	Low	Low	Low
Bronzan et al. (2007) [35]	High	Low	Low	Low	Low	High	Low	Low	Low
Issifou et al. (2007) [36]	High	Low	Low	Low	Low	Unclear	Low	Low	Low
Oduro et al. (2007) [37]	High	Low	Low	Low	Low	Low	Unclear	Unclear	Low
Orimadegun et al. (2007) [38]	High	Unclear	Unclear	Low	Low	Low	Low	Low	Low
Bassat et al. (2008) [39]	Low	Low	Low	Low	Low	Low	Low	Low	Low
Ranque et al. (2008) [40]	High	Low	Low	Low	Low	High	Low	Low	Low
Ogetii et al. (2010) [41]	Low	NA	Low	Low	Low	Low	NA	Low	Low
Camara et al. (2011) [42]	Low	Unclear	Unclear	Low	Low	Low	Low	Unclear	Low
Hendriksen et al. (2012) [15]	Low	NA	Low	Low	Low	Low	NA	Low	Low
Hendriksen et al. (2012) [14]	Low	Low	Low	Low	Low	Low	Low	Low	Low
Jallow et al. (2012) [18]	Low	Low	Low	Low	High	Low	Low	Low	Low
von Seidlein et al. (2012) [13]	Low	Low	Low	Low	Low	Low	Low	Low	Low
Kendjo et al. (2012) [19]	Low	Low	Low	Low	High	High	Low	Low	Low
Orimadegun et al. (2014) [43]	Low	Low	Low	Low	Low	Low	Low	Low	Low

*NA* not applicable

**Fig. 2:**
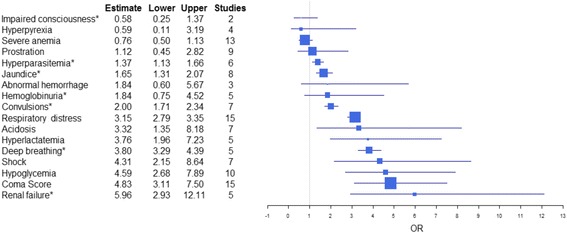
Pooled estimates of odds ratios (with 95% confidence intervals) of each predictor of mortality assessed in at least two studies (without the Severe Malaria in African Children studies) and number of studies by each predictor. The size of each predictor’s box is proportional to the global sample size of studies involved in the corresponding summary odds ratios. *Results calculated by fixed effects

**Fig. 3:**
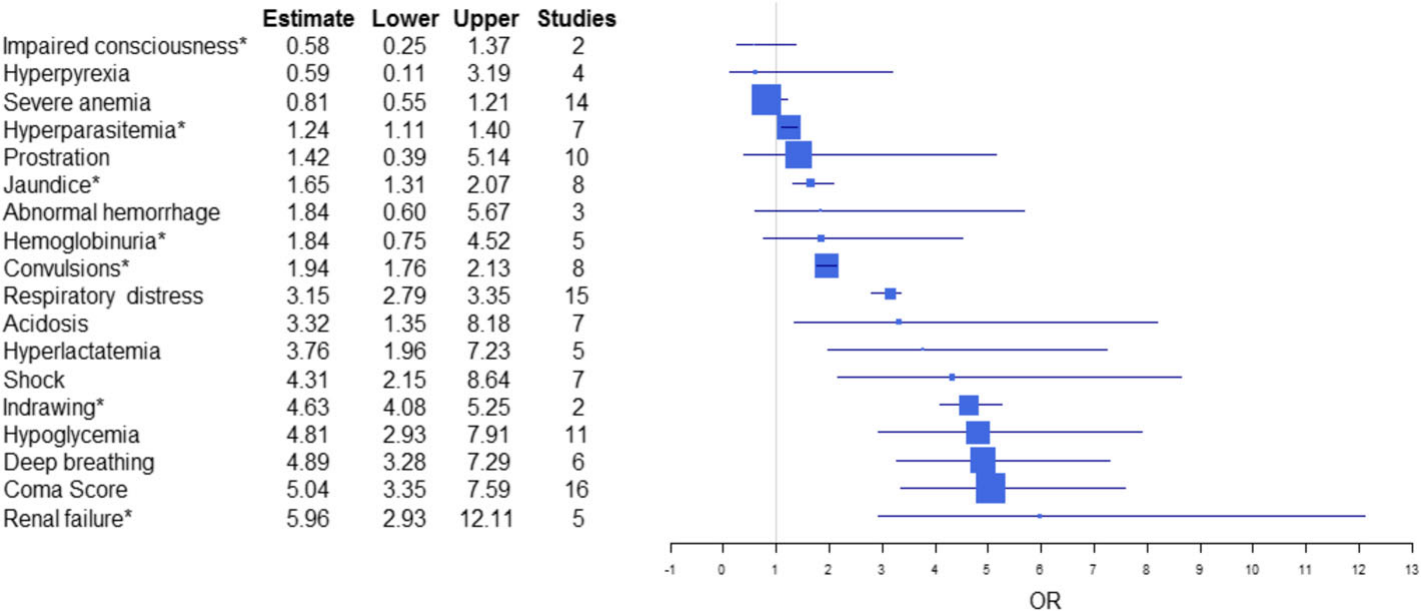
Pooled estimates of odds ratios (with 95% confidence intervals) of each predictor of mortality assessed in at least two studies (including the Severe Malaria in African Children studies) and number of studies by each predictor. The size of each predictor’s box is proportional to the global sample size of studies involved in the corresponding summary odds ratios. *Results calculated by fixed effects

A total of 36 different prognostic indicators associated with death due to severe malaria were identified in 30 studies. The number of predictors of mortality evaluated per study ranged from 1 to 19 (median 6.5, interquartile range 3–11). Out of 36 identified prognostic indicators, 18 corresponded with the clinical criteria of severe malaria established by the WHO. Two forest plots displaying pooled estimates of ORs with 95% confidence intervals (CI) calculated for 17 and 18 prognostic indicators included in the WHO definition of severe malaria are captured in Figs. 2 and 3, respectively. Definitions and further characteristics of the analyzed prognostic indicators are assembled in Table 3.

**Table 3:** Characteristics of assessed WHO prognostic indicators

Predictor of mortality	Definition	N assessed	N with the condition	N died	Pooled PPV	Odds ratio (meta-analysis)	Study reference
Neurological symptoms and signs							
Convulsions	≥2/24 hours	8197	2252	772	14.7%	2 (1.71–2.34)	von Seidlein 2012 [13]; Bassat 2008 [39]; Mockenhaupt 2004 [30]; Imbert 2003 [28]; Gay-Andrieu 2005 [32]; Camara 2011 [42]; Modiano 1998 [25]
Convulsions^a^	≥2/24 hours	34233	10573	1901	8.1%	1.94 (1.76–2.13)	von Seidlein 2012 [13]; Bassat 2008 [39]; Mockenhaupt 2004 [30]; Imbert 2003 [28]; Gay-Andrieu 2005 [32]; Camara 2011 [42]; Modiano 1998 [25]; Kendjo 2013 [19]
Coma score	BCS ≤2	16796	1675	4881	21.3%	4.83 (3.11–7.5)	Krishna 1994 [23]; von Seidlein 2012 [13]; Ranque 2008 [40]; Orimadegun 2007 [38]; Oduro 2007 [37]; Mockenhaupt 2004 [30]; Jallow 2012 [18]; Issifou 2007 [36]; Gay-Andrieu 2005 [32]; English 1997 [17]; Dzeing-Ella 2005 [31]; Bassat 2008 [39]; Camara 2011 [42]; Bronzan 2007 [35]; Orimadegun 2014 [43]
Coma score^a^	BCS ≤2	42832	7316	2804	20.7%	5.04 (3.35–759)	Krishna 1994 [23]; von Seidlein 2012 [13]; Ranque 2008 [40]; Orimadegun 2007 [38]; Oduro 2007 [37]; Mockenhaupt 2004 [30]; Jallow 2012 [18]; Issifou 2007 [36]; Gay-Andrieu 2005 [32]; English 1997 [17]; Dzeing-Ella 2005 [31]; Bassat 2008 [39]; Camara 2011 [42]; Bronzan 2007 [35]; Orimadegun 2014 [43]; Kendjo 2013 [19]
Impaired consciousness	BCS 3–4	276	95	42	9.5%	0.58 (0.25–1.37)	Gay-Andrieu 2005 [32]; Camara 2011 [42]
Prostration	Cannot sit OR cannot eat (breastfeed) OR cannot walk OR cannot stand up	11607	6452	1176	8.3%	1.12 (0.45–2.82)	Jallow 2012 [18]; Zeidan 2005 [34]; von Seidlein 2012 [13]; Mockenhaupt 2004 [30]; Imbert 2003 [28]; Camara 2011 [42]; Bassat 2008 [39]; Assimadi 1998 [24]; Modiano 1998 [25]
Prostration^a^	Cannot sit OR cannot eat (breastfeed) OR cannot walk OR cannot stand up	37643	22322	2305	7.0%	1.42 (0.39–5.14)	Jallow 2012 [18]; Zeidan 2005 [34]; von Seidlein 2012 [13]; Mockenhaupt 2004 [30]; Imbert 2003 [28]; Camara 2011 [42]; Bassat 2008 [39]; Assimadi 1998 [24]; Modiano 1998 [25]; Kendjo 2013 [19]
Respiratory symptoms and signs							
Deep breathing	NR	9106	1230	965	22.2%	3.8 (3.29–4.39)	von Seidlein 2012 [13]; Maitland 2003 [29]; English 1996 [16]; Varandas 2000 [26]; Jallow 2012 [18]
Deep breathing^a^	NR	32882	4049	1772	19.1%	4.89 (3.28–7.29)	von Seidlein 2012 [13]; Maitland 2003 [29]; English 1996 [16]; Varandas 2000 [26]; Jallow 2012 [18]; Kendjo 2013 [19]
Indrawing^a^	NR	26386	3137	1159	13.3%	4.63 (4.08–5.25)	English 1996 [16]; Kendjo 2013 [19]
Respiratory distress	Nasal flaring or costal indrawing or accessory muscle use OR Kussmal breathing/Cheyne–Stokes breathing OR deep breathing OR abnormalities in respiratory rate/rhythm OR dyspnea OR pulmonary edema	15343	3729	1526	17.5%	3.15 (2.79–3.35)	Jallow 2012 [18]; von Seidlein 2012 [13]; Ranque 2008 [40]; Oduro 2007 [37]; Mockenhaupt 2004 [30]; Issifou 2007 [36]; Imbert 2003 [28]; Gay Andrieu 2005 [32]; English 1996 [16]; Dzeing-Ella 2005 [31]; Camara 2011 [42]; Bassat 2008 [39]; Assimadi 1998 [24]; Varandas 2000 [26]; Modiano 1998 [25]
Cardiovascular symptoms and signs							
Shock/circulatory collapse	SBP <50 OR compensated shock (BP ≥70 + CRT ≥3 s) and decompensated (BP <70) OR SBP <60 in ≤5-year-old children OR SBP <80 in >5-year-old children OR septic shock score ≥2	7567	915	789	23.6%	4.31 (2.15–8.64)	Jallow 2012 [18]; von Seidlein 2012 [13]; Mockenhaupt 2004 [30]; Maitland 2003 [29]; Imbert 2003 [28]; Assimadi 1998 [24]; Camara 2011 [42]
Abnormalhemorrhage	NR	1349	17	199	23.5%	1.84 (0.6–5.67)	Assimadi 1998 [24]; Modiano 1998 [25]
Jaundice							
Jaundice		11178	599	1203	17.4%	1.65 (1.31–2.07)	Jallow 2012 [18]; von Seidlein 2012 [13]; Mockenhaupt 2004 [30]; Maitland 2003 [29]; Imbert 2003 [28]; Camara 2011 [42]; Bassat 2008 [39]; Assimadi 1998 [24]
Renal symptoms and signs							
Hemoglobinuria	Verified by dipstick	7102	286	739	11.5%	1.84 (0.75–4.52)	Mockenhaupt 2004 [30]; Imbert 2003 [28]; Assimadi 1998 [24]; Camara 2011 [42]; Orimadegun 2014 [43]; von Seidlein 2012 [13]
Laboratory values of severe malaria in selected studies							
Acidosis	BE < –8 mmol/L OR base deficit >15 OR BE < –12 mmol/L (all)	6549	2392	646	15.1%	3.32 (1.35–8.18)	Hendriksen 2012 [14]; Maitland 2005; Jallow 2012 [18]; Maitland 2003 [29]; English 1996 [16], Assimadi 1998 [24]; Orimadegun 2014 [43]
Severe anemia	Hematocrit <15% or Hb <5 g/dL	14078	5039	1406	8.2%	0.76 (0.50–1.13)	Jallow 2012 [18]; Hendriksen 2012 [14]; Camara 2011 [42]; Bassat 2008 [39]; Ranque 2008 [40]; Orimadegun 2007 [38]; Mockenhaupt 2004 [30]; Maitland 2003 [29]; Issifou 2007 [36]; Gay-Andrieu 2005 [32]; Dzeing-Ella 2005 [31]; Imbert 2003 [28]; Modiano 1998 [25];
Severe anemia^a^	Hematocrit <15% or Hb <5 g/dL	40114	10418	2535	7.4%	0.81 (0.55–1.21)	Jallow 2012 [18]; Hendriksen 2012 [14]; Camara 2011 [42]; Bassat 2008 [39]; Ranque 2008 [40]; Orimadegun 2007 [38]; Mockenhaupt 2004 [30]; Maitland 2003 [29]; Issifou 2007 [36]; Gay-Andrieu 2005 [32]; Dzeing-Ella 2005 [31]; Imbert 2003 [28]; Modiano 1998 [25]; Kendjo 2013 [19]
Hyperlactatemia	Lactate ≥5 mmol/L or NR	2188	773	183	14.7%	3.76 (1.96–7.23)	Jallow 2012 [18]; Krishna 1994 [23]; Oduro 2007 [37]; Mockenhaupt 2004 [30]; Dzeing-Ella 2005 [31]
Hyperparasitemia	Hyperparsitemia >10% ([14]) or hyperparasitemia >4% ([32]) or parasitemia ≥250,000 p/μL ([43]) or *P. falciparum* parasite density >500,000/μL ([18])	7735	1164	873	13.2%	1.37 (1.13–1.66)	Jallow 2012 [18]; Hendriksen 2012 [14]; Mockenhaupt 2004 [30]; Imbert 2003 [28]; Gay-Andrieu 2005 [32]; Orimadegun 2014 [43]
Hyperparasitemia^a^	Hyperparsitemia > 10% ([14]) or hyperparasitemia >4% ([32]) or parasitemia ≥250,000 p/μL ([19, 43]) or *P. falciparum* parasite density >500,000/μL ([18])	33771	6123	2002	6.5%	1.24 (1.11–1.4)	Jallow 2012 [18]; Hendriksen 2012 [14]; Mockenhaupt 2004 [30]; Imbert 2003 [28]; Gay-Andrieu 2005 [32]; Orimadegun 2014 [43]; Kendjo 2013 [19]
Hypoglycemia	Glucose <2.2 mmol/L	6358	37	48	16.2%	4.59 (2.68–7.89)	Jallow 2012 [18]; Krishna 1994 [23]; Imbert 2003 [28]; Mockenhaupt 2004 [30]; Issifou 2007 [36]; Gay-Andrieu 2005 [32]; Dzeing-Ella 2005 [31]; Camara 2011 [42]; Bassat 2008 [39]; Modiano 1998 [25]
Hypoglycemia^a^	Glucose <2.2 mmol/L	31348	2933	2662	45.4%	4.81 (2.93–7.91)	Jallow 2012 [18]; Krishna 1994 [23]; Imbert 2003 [28]; Mockenhaupt 2004 [30]; Issifou 2007 [36]; Gay-Andrieu 2005 [32]; Dzeing-Ella 2005 [31]; Camara 2011 [42]; Bassat 2008 [39]; Modiano 1998 [25]; Kendjo 2013 [19]
Renal failure	Urine output of <12 mL/kg/24 hours and serum creatinine >265 μL/L over OR plasma creatinine >3 mg/dL	4757	32	547	40.6%	5.96 (2.93–12.11)	Jallow 2012 [18]; Imbert 2003 [28]; Assimadi 1998 [24]; Modiano 1998 [25]; Orimadegun 2014 [43]
Other symptoms and signs							
Hyperpyrexia	>40 °C OR ≥40 °C	3946	125	919	24.8%	0.59 (0.11–3.19)	Jallow 2012 [18]; Zeidan 2005 [34]; Mockenhaupt 2004 [30]; Imbert 2003 [28]

^a^Including the Severe Malaria in African Children studies.

*BE* base excess, *BCS* Blantyre coma scale, *BP* blood pressure, *CRT* capillary refill time, *HB* hemoglobin, *NR* not reported, *PPV* positive predictive value, *SBP* systolic blood pressure

Prognostic indicators with the strongest association with death included renal failure (5.96, 95% CI 2.93–12.11), coma (4.83, 95% CI 3.11–7.5), hypoglycemia (4.59, 95% CI 2.68–7.89), shock (4.31, 95% CI 2.15–8.64), and deep breathing (3.8, 95% CI 3.29–4.39). These five indicators also had the largest CI boundaries. Respiratory distress, while having a lower OR than the five indicators mentioned above, presented a narrower CI and lower CI boundaries in line with five top indicators (3.15, 95% CI 2.79–3.35). Moreover, the results were also consistent upon introduction of the SMAC study, with each association being slightly larger than without the SMAC, while the association with death of the top indicators was more homogeneous for renal failure (5.96, 95% CI 2.93–12.11), coma (5.04, 95% CI 3.35–7.59), deep breathing 4.89 (95% CI 3.28–7.29), hypoglycemia (4.81, 95% CI 2.93–7.91), and chest indrawing (4.63, 95% CI 4.08–5.25). The latter entered the top five indicators (in place of shock) and also presented the lower CI boundary (>4).

Two or more convulsions (2.0, 95% CI 1.71–2.34) were also associated with poor outcome. However, further neurological signs, such as prostration (1.12, 95% CI 0.45–2.82) and impaired consciousness (0.58, 95% CI 0.25–1.37) were not associated with death. These results are comparable to those after inclusion of the SMAC study, namely convulsions (1.94, 95% CI 1.76–2.13) and prostration (1.42, 95% CI 0.39–5.14). Neither severe anemia, with and without the SMAC studies (0.81, 95% CI 0.55–1.21 vs. 0.76, 95% CI 0.50–1.13, respectively) nor hyperpyrexia (1.19, 95% CI 0.71–1.99) were associated with death.

## Discussion

The results of the meta-analysis show that there is a large variation in the strength of the association between the different WHO-defined criteria of severe malaria and death. Renal failure, coma, hypoglycemia, shock, and respiratory distress represent those with the highest prognostic value. These manifestations were also those with the highest prognostic value for death in the original paper by Marsh [12], which was supportive of the WHO definition of severe malaria. Similarly, impaired consciousness, prostration, hyperpyrexia, hyperparasitaemia, and severe anemia were weak predictors both in the present systematic review and in Marsh’s paper [12]. While 5039 (35.7%) of children from the enclosed studies suffered from severe anemia, its association with death, though widely acknowledged, was insignificant. This can possibly be explained by the fact that anemic children receive blood transfusion upon admission or by the lack of other concomitant feature such as respiratory distress or neurological impairment. On the other hand, hypoglycemia, which similarly to severe anemia could be reversed if early detected, remains a significant marker of severity, which can be possibly explained by its dependency on other severe markers. Conditions such as malnutrition or HIV co-infection have not been addressed in this analysis since they are not part of the definition of severe malaria. They are, however, very important contributors of mortality and should definitively be considered together with other clinical features when assessing a sick child.

The current systematic review recognizes coma (defined as Blantyre coma scale (BCS) ≤ 2) and deep breathing as robust prognostic factors of pediatric life-threatening malaria that can simply be determined and recorded by skilled observers in all types of settings. Deep breathing, as a crucial respiratory sign of severe malaria, is commonly a compensatory manifestation of underlying metabolic acidosis [44] and is more predictive than respiratory distress accompanied by signs of variable severity. These findings are nearly in line with the results from a prospective study [12] of 1844 patients in Kenya, which identified respiratory distress and impaired consciousness (defined as prostration or coma) as highly associated with death and, except for prostration, with the Lambaréné Organ Dysfunction Score, which combines coma, prostration, and deep breathing [10].

Although there is no definite consensus regarding the strongest predictors of death within the WHO clinical definition of severe malaria, the WHO distinguished three groups [1] classing clinical and laboratory features of the disease in a way to facilitate appropriate treatment. A major contrast of our results with the clinical features included in the WHO Group 1 symptoms (prostrate but conscious, prostrate with impaired consciousness, coma, mild/severe respiratory distress, shock), which are supposedly more severe and for which parenteral treatment is recommended, is that a child with prostration or impaired consciousness appears to be at a low risk of death when compared with the presence of any other listed signs and symptoms. One possible explanation for this unexpected finding is that, in some studies, the definition of impaired consciousness was less stringent than that of the WHO (BCS < 3). Interestingly, in the differentiated group of 1289 Gabonese children, Issifou et al. [36] applied a BCS between 3 and 4 to classify cases of moderate malaria. On the other hand, our findings are consistent with the WHO Group 2 clinical features (severe anemia, two or more convulsions in past 24 hours, hemoglobinuria, jaundice), which indicate a disease of lower severity and for which a supervised oral therapy is recommended.

The present attempt to rank clinical features according to their prognostic values was performed to potentially better distinguish children that should definitely be receiving parenteral treatment versus those that could be considered for prompt oral treatment with artemisinin-based combinations. At present, the WHO recommends injectable artesunate for all children with asexual forms of *P. falciparum* in peripheral blood and at least one criterion of severity [45]. In the light of the very different prognostic values of the different features, Kopel et al. [46] suggested that oral treatment could be a successful alternative for patients with a detected parasitemia and a criterion considered as less severe, e.g., jaundice. Certainly, all prognostic indicators that are able to be detected at the bedside need to be searched for, and finding a low-prognostic symptom or sign does not remove the need for parenteral treatment if a high-prognostic one is present. Identifying a subset of patients with moderately severe malaria who could be safely managed with oral treatment at the primary care level would simplify the patients’ management in settings where referral to hospital for injectable treatment is difficult, and allow better resources allocation. A simplified approach may be easier to implement. Already, in settings where laboratory facilities are unavailable, the laboratory tests used to define severe malaria are not considered in the classification of the disease. This new approach should be carefully assessed in a prospective multicentric clinical trial to demonstrate its safety.

To our knowledge, this is the first systematic review and meta-analysis of predictors of death drawn from all relevant studies of African children with strictly defined severe malaria. Methodological quality was assessed by using a priori adjusted and defined rules of the latest version of QUADAS-2 tool, which allowed better evaluation of risk of biases in several domains. In addition, this review assessed the disease severity criteria used in the SMAC studies [19]. Indeed, this represents the largest sample size ever recruited. The fact that the results did not change much when including or not prognostic indicators from the SMAC studies increases the robustness of the findings.

The main limitation of our analysis comes from the methodological or reporting weaknesses of some studies, of which the most important one is the lack of reproducibility of reported clinical symptoms and signs. Indeed, the inter-observer (clinician) agreement on the assessment of some of the signs, such as impaired consciousness or prostration for example, can be very low. Additionally, heterogeneity between studies regarding availability of laboratory data, threshold used to define abnormality, and quality of healthcare, especially with regards to blood transfusion and management of renal failure, need to be taken into account in results interpretation. Another limitation of our review is that it did not consider combinations of clinical and laboratory features of severe malaria because of the unavailability of individual records. It has been shown that having more than one manifestation of severe malaria increases the risk of dying [13] and this has to be taken into account in a child assessment of severity, and hence in case management. Furthermore, due to lack of data in the included studies, this meta-analysis could not explore the impact of other concurrent complications that do not form part of the definition of severe malaria but are known for increasing the risk of death such as, for example, bacteremia. In addition, since all data were aggregated in each study, we were not able to analyze predictors by age group or sex. This should not alter much the relevance of our findings since approximately 80% patient population was < 5 years of age and WHO has never considered a differential definition of severe malaria for children and adults or male and female. Finally, studies reporting less than 100 cases were excluded to reduce complexity, but some of those could have brought relevant information.

## Conclusion

In conclusion, the findings of this meta-analysis show that the strength of association between the criteria defining severe malaria and death is quite variable for each clinical and/or laboratory features (OR ranging from 0.58 to 5.96). Despite the heterogeneity of entry criteria, the individual studies provided concordant results. A ranking allowed the identification of features weakly associated with death, such as impaired consciousness and prostration, which could assist to refine case definition and thus optimize antimalarial treatment.

### Additional files

Additional file 1: Table S1. QUADAS-2 review-specific tailored tool and instructions for quality assessment of selected studies. (DOCX 19 kb)

Additional file 2: QUADAS-2 tool. Risk of bias and applicability judgments. (PDF 499 kb)

## Appendix

### Criteria for study selection

Design: Epidemiological, clinical, and treatment studies on malaria were selected. Controlled trials, non-controlled trials, cohort studies, case control studies, and case series, either prospective or retrospective of 100 cases or more, were accepted. Systematic reviews, meta-analyses, letters, editorials, and comments were used only as a source of reference.

Setting: Studies conducted in Africa.

Participants: Studies that included children below the age of 15 with severe malaria according to the WHO definitions, with or without modified criteria. The cases needed to be diagnosed with parasitological confirmation defined as parasite identification by smear or as a positive rapid diagnostic test.

Outcome: Studies that reported death rate.

Prognostic indicators: Studies that reported clinical and laboratory variables associated with death due to malaria.

Reference standard: any reported death from admission to end of follow-up.

Data reporting: Studies that allow the reconstruction of two-by-two tables made up of death rate and prognostic indicators of death. When necessary, authors were contacted to obtain missing data.

## Abbreviations

BCS: Blantyre Coma Scale
CI: confidence interval
ORs: odds ratios
PRISMA: Preferred Reporting Items for Systemic Reviews and Meta-Analyses
QUADAS: Quality Assessment of Diagnostic Accuracy Studies
SMAC: Severe Malaria in African Children
WHO: World Health Organization
