# Supplementary material for: Clinical and laboratory predictors of death in African children with features of severe malaria: a systematic review and meta-analysis
Source: BMC Med. 2017 Aug 3;15:147. doi: 10.1186/s12916-017-0906-5 (PMC5541406; doi:10.1186/s12916-017-0906-5)
Supplement: Supplementary file 1 — QUADAS-2 review-specific tailored tool and instructions for quality assessment of selected studies. (DOCX 19 kb) [file 12916_2017_906_MOESM1_ESM.docx]

**Table S1. QUADAS-2 review-specific tailored tool and instructions for quality assessment of selected studies**

| **Domain 1 : PATIENT SELECTION**   1. **Risk of Bias**   **Description:**  Describe methods of patient selection:  Q1. Was a consecutive or random sample of patients enrolled?  *Yes: ‘all children’*  Q2. Was a case-control design avoided?  Q.3 Did the study avoid inappropriate exclusions?  *Exclusions considered as inappropriate: e.g. patients difficult to diagnose, patients with bad outcome*  **Applicability:** Is there concern that the included patients do not match the review question?  *Match the review question: children <15 years old with severe malaria according to WHO definitions, with or without modified criteria, diagnosed with parasitological confirmation*  *High concern: restrictive clinical criteria used as inclusion (only cerebral malaria and malarial anemia etc.), age limit leading to inclusion of population of higher risk of dying (e.g. children aged 6-102 months), or presence of another disease (e.g. sepsis, pneumonia, meningitis)* |
| --- |

| **Domain 2: INDEX TESTS** (suggestion to classify and assess lab values and clinical values as separate groups)   1. **Risk of Bias**   **Description:** Describe the index test and how it was conducted and interpreted:  Q1. Were the index test results interpreted without knowledge of the reference standard?  Q2. Were the index test results collected prospectively?  Q3. Were the index test results established and reported using standardized clinical procedures (make attention) and data collection tools?  *Risk of bias HIGH: if retrospective analyses of unstandardized medical forms, if only preselected index tests considered*  **Applicability:** Is there concern that the index test, its conduct, or interpretation differs from the review question?  *The review question is not restrictive to any particular type of index test.* |
| --- |

| **DOMAIN 3: REFERENCE STANDARD**   1. **Risk of Bias**   **Description:** Describe the reference standard and how it was conducted and interpreted:  *Any reported death was considered to be a reference standard*  Q1. Is the reference standard likely to correctly classify the target condition?  Q2. Was the reference standard result interpreted without knowledge of the index test?  **Risk of bias:**  Could the reference standard, its conduct, or its interpretation have introduced bias?  **Applicability:** Is there concern that the target condition as defined by the reference standards does not match the review question? |
| --- |

| **DOMAIN 4: FLOW AND TIMING**   1. **Risk of Bias**   **Description:**  Describe any patients who did not receive the index test(s) and/or reference standard or who were excluded from the 2x2 table (refer to flow diagram):  Describe the time interval and any interventions between index test(s) and reference standard:  *(give hours, days, in any case precise the time interval)*  Q1. Was there an appropriate interval between index test(s) and reference standard?  *Any time interval between index test and death was considered appropriate*  Q2. Did all patients receive reference standard?  *Reference standard (death) is an inclusion criteria for selected studies*  Q3. Did patients receive the same reference standard?  *Yes: any death reported*  Q4. Were all patients included in the analysis?  *Yes: At least 80% of enrolled patients should be included in the analysis*  *A potential for bias exists if number of patients enrolled differs from the number of patients ultimately included and lost for reasons such as: e.g. unknown reasons, development of neurological sequel*  **Risk of bias:** Could the patient flow have introduced bias?  *Risk of bias HIGH: no to Q4* |
| --- |
