# Supplementary material for: Clinical and laboratory predictors of death in African children with features of severe malaria: a systematic review and meta-analysis
Source: BMC Med. 2017 Aug 3;15:147. doi: 10.1186/s12916-017-0906-5 (PMC5541406; doi:10.1186/s12916-017-0906-5)
Supplement: Supplementary file 2 — QUADAS-2 tool. Risk of bias and applicability judgments. (PDF 499 kb) [file 12916_2017_906_MOESM2_ESM.pdf]

QUADAS-2 tool: Risk of bias and applicability judgments   Study reference: KRISHNA 1994

Domain 1: Patient selection

A. Risk of bias

Describe methods of patient selection:

Children aged 18 months-12 years, with severe faciparum malaria(confirmed by blood film exam),all children had 1≥ feature: BCS≤2, parasitemia >100000/µL with 15%, or shock. Exclusion criteria: chloroquine treatment in last 4h, other causes of fever or altered consciousness (examination, blood/CSF culture)

- Was a consecutive or random sample of patients enrolled? Yes
- Was a case-control design avoided? Yes
- Did the study avoid inappropriate exclusions? Yes

Could the selection of patients have introduced bias? **RISK: LOW**

B. Concerns regarding applicability

Describe included patients (prior testing, presentation, intended use of index test and setting):

See above

Is there concern that the included patients do not match the review question? **CONCERN: HIGH**

Domain 2a: Laboratory index tets: lactatemia and glycemia

A. Risk of bias

Describe the index test and how it was conducted and interpreted:

Blood glucose level and packed cell volume measured immediately;baseline blood samples for assessment of glucose, lactate

- Were the index test results interpreted without knowledge of the results of the reference standard? Yes
- Were the index test results collected prospectively? Yes
- Were the index test results established and reported using standarized clinical procedures and data collection tools? Yes

Could the conduct or interpretation of the index test have introduced bias? **RISK: LOW**

B. Concerns regarding applicability

Is there concern that the index test, its conduct, or interpretation differ from the review question **CONCERN: LOW**

Domain 2b: Clinical index tests: coma score

A. Risk of bias

Describe the index test and how it was conducted and interpreted:

BCS≤2 on admission; if a history of recent convulsion (<1 h before admission) or convulsion on admission, coma score evaluated 30 minutes after the last convulsion

• Were the index test results interpreted without knowledge of the results of the reference standard?	Yes
• Were the index test results collected prospectively?	Yes
• Were the index test results established and reported using standarized clinical procedures and data collection tools?	Yes
**Could the conduct or interpretation of the index test have introduced bias?**	**RISK: LOW**

B. Concerns regarding applicability

**Is there concern that the index test, its conduct, or interpretation differ from the review question?**	**CONCERN: LOW**

Domain 3: Reference standard

A. Risk of bias

Describe the reference standard and how it was conducted and interpreted:

Any reported death was considerd to be a reference standard

• Is the reference standard likely to correctly classify the target condition?	Yes
• Were the reference standard results interpreted without knowledge of the results of the index test?	Yes
**Could the reference standard, its conduct, or its interpretation have introduced bias?**	**RISK: LOW**

B. Concerns regarding applicability

**Is there concern that the target condition as defined by the reference standard does not match the review question?**	**CONCERN: LOW**

Domain 4: Flow and timing

A. Risk of bias

Describe any patients who did not receive the index test(s) and/or reference standard or who were excluded from the 2x2 table (refer to flow diagram):

One of excluded patients died immediately on arrival before recieving treatment

Describe the time interval and any interventions between index test(s) and reference standard:

Median time to death 18.5 h

• Was there an appropriate interval between index test(s) and reference standard?	Yes
• Did all patients receive a reference standard?	Yes
• Did patients receive the same reference standard?	Yes
• Were all patients included in the analysis?	Yes
**Could the patient flow have introduced bias?**	**RISK: LOW**

Domain 1: Patient selection

A. Risk of bias

Describe methods of patient selection: *All children with positive blood film for P. falciparum and 1≥ of clinical features: coma or prostration, hyperparasitemia, respiratory distress*

- Was a consecutive or random sample of patients enrolled? Yes
- Was a case-control design avoided? Yes
- Did the study avoid inappropriate exclusions? Yes

Could the selection of patients have introduced bias? **RISK: LOW**

B. Concerns regarding applicability

Describe included patients (prior testing, presentation, intended use of index test and setting):   *See above*

Is there concern that the included patients do not match the review question? **CONCERN: LOW**

Domain 2a: Clinical index tests: respiratory distress, deep breathing, nasal flaring, indrawing

A. Risk of bias

Describe the index test and how it was conducted and interpreted: *On admission clinicians indicated on a checklist respiratory symptoms and signs, including signs such as: nasal flaring, indrawing, deep breathing or signs of pulmonary edema*

- Were the index test results interpreted without knowledge of the results of the reference standard? Yes
- Were the index test results collected prospectively? Yes
- Were the index test results established and reported using standarized clinical procedures and data collection tools? Yes

Could the conduct or interpretation of the index test have introduced bias? **RISK: UNCLEAR**

B. Concerns regarding applicability

Is there concern that the index test, its conduct, or interpretation differ from the review question? **CONCERN: LOW**

Domain 2b: Laboratory index tets: acidosis

A. Risk of bias

Describe the index test and how it was conducted and interpreted:
Among all children with respiratory distress (119) an arterial blood gas sample was taken within 4h of admission in 61% of them; procedure not reported in case of othe children

• Were the index test results interpreted without knowledge of the results of the reference standard?	Yes
• Were the index test results collected prospectively?	Yes
• Were the index test results established and reported using standarized clinical procedures and data collection tools?	Yes
**Could the conduct or interpretation of the index test have introduced bias?**	**RISK: LOW**

B. Concerns regarding applicability

**Is there concern that the index test, its conduct, or interpretation differ from the review question?**	**CONCERN: LOW**

Domain 3: Reference standard

A. Risk of bias

Describe the reference standard and how it was conducted and interpreted:
Any reported death was considerd to be a reference standard

• Is the reference standard likely to correctly classify the target condition?	Yes
• Were the reference standard results interpreted without knowledge of the results of the index test?	Yes
**Could the reference standard, its conduct, or its interpretation have introduced bias?**	**RISK: LOW**

B. Concerns regarding applicability

**Is there concern that the target condition as defined by the reference standard does not match the review question?**	**CONCERN: LOW**

Domain 4: Flow and timing

A. Risk of bias

Describe any patients who did not receive the index test(s) and/or reference standard or who were excluded from the 2x2 table (refer to flow diagram):
Children admitted in critical condition who died before the admission; other excluded if historical data or investigation indicated on presence of another significant pathology: lobar pneumonia on CXR, septicemia, meningitis, accidental poisoning, congenital heart, renal disease, preceding developmental delay, epilepsy
Describe the time interval and any interventions between index test(s) and reference standard:
NR

• Was there an appropriate interval between index test(s) and reference standard?	Yes
• Did all patients receive a reference standard?	Yes
• Did patients receive the same reference standard?	Yes
• Were all patients included in the analysis?	Yes
**Could the patient flow have introduced bias?**	**RISK: LOW**

QUADAS-2 tool: Risk of bias and applicability judgments   Study reference: ENGLISH 1997

Domain 1: Patient selection

A. Risk of bias

Describe methods of patient selection:

All children with positive blood film for P. falciparum and 1≥ of clinical features: coma or prostration, hyperparasitemia, respiratory distress

• Was a consecutive or random sample of patients enrolled?	Yes
• Was a case-control design avoided?	Yes
• Did the study avoid inappropriate exclusions?	No
**Could the selection of patients have introduced bias?**	**RISK: HIGH**

B. Concerns regarding applicability

Describe included patients (prior testing, presentation, intended use of index test and setting):

See above

**Is there concern that the included patients do not match the review question?**	**CONCERN: UNCLEAR**

Domain 2: Clinical index test

A. Risk of bias

Describe the index test and how it was conducted and interpreted:

BCS <3 at least 30 min after the last seizure, at least 6 h after diazepam treatment and treatment of hypoglycemia if appropriate

• Were the index test results interpreted without knowledge of the results of the reference standard?	Yes
• Were the index test results collected prospectively?	Yes
• Were the index test results established and reported using standarized clinical procedures and data collection tools?	Yes
• If a treshold was used was it pre-specified?	Yes
**Could the conduct or interpretation of the index test have introduced bias?**	**RISK:LOW**

B. Concerns regarding applicability

**Is there concern that the index test, its conduct, or interpretation differ from the review question?**	**CONCERN:HIGH**

Domain 3: Reference standard

A. Risk of bias

Describe the reference standard and how it was conducted and interpreted:
Any reported death was considerd to be a reference standard

- Is the reference standard likely to correctly classify the target condition? — Yes
- Were the reference standard results interpreted without knowledge of the results of the index test? — Yes

Could the reference standard, its conduct, or its interpretation have introduced bias? **RISK: LOW**

B. Concerns regarding applicability

Is there concern that the target condition as defined by the reference standard does not match the review question? **CONCERN: LOW**

Domain 4: Flow and timing

A. Risk of bias

Describe any patients who did not receive the index test(s) and/or reference standard or who were excluded from the 2x2 table (refer to flow diagram):
Children admitted in critical condition who died before the admission; other excluded if historical data or investigation indicated on presence of another significant pathology: lobar pneumonia on CXR, septicemia, meningitis, accidental poisoning, congenital heart, renal disease, preceding developmental delay, epilepsy
Describe the time interval and any interventions between index test(s) and reference standard:
NR

- Was there an appropriate interval between index test(s) and reference standard? — Yes
- Did all patients receive a reference standard? — Yes
- Did patients receive the same reference standard? — Yes
- Were all patients included in the analysis? — Yes

Could the patient flow have introduced bias? **RISK: LOW**

QUADAS-2 tool: Risk of bias and applicability judgments   Study reference: ASSIMADI 1998

Domain 1: Patient selection

A. Risk of bias

Describe methods of patient selection:<br>*All children with severe malaria according to WHO definition (1995)*

- Was a consecutive or random sample of patients enrolled? Yes
- Was a case-control design avoided? Yes
- Did the study avoid inappropriate exclusions? Yes

Could the selection of patients have introduced bias? **RISK: LOW**

B. Concerns regarding applicability

Describe included patients (prior testing, presentation, intended use of index test and setting):<br>*See above*

Is there concern that the included patients do not match the review question? **CONCERN: LOW**

Domain 2: Clinical index tests

A. Risk of bias

Describe the index test and how it was conducted and interpreted:<br>*No fixed coma score applied, instead impaired consciousness: children who wake up upon a stimulus, but fall asleep immediately, coma: more severe than impaired consciousness. Respiratory distress: any cause other than anemia, possible causes: convulsive crises, accidental drugs inhalation, acute pulmonary edema, respiratory infections, failure of automatic control of breathing*

- Were the index test results interpreted without knowledge of the results of the reference standard? Yes
- Were the index test results collected prospectively? Yes
- Were the index test results established and reported using standarized clinical procedures and data collection tools Unclear

Could the conduct or interpretation of the index test have introduced bias? **RISK:HIGH**

B. Concerns regarding applicability

Is there concern that the index test, its conduct, or interpretation differ from the review question? **CONCERN:HIGH**

Domain 2b: Laboratory index tests

A. Risk of bias

Describe the index test and how it was conducted and interpreted:
NR

- Were the index test results interpreted without knowledge of the results of the reference standard? Yes
- Were the index test results collected prospectively? Yes
- Were the index test results established and reported using standarized clinical procedures and data collection tools? Unclear
- If a treshold was used was it pre-specified? No

Could the conduct or interpretation of the index test have introduced bias? **RISK:HIGH**

B. Concerns regarding applicability

Is there concern that the index test, its conduct, or interpretation differ from the review question? **CONCERN: HIGH**

Domain 3: Reference standard

A. Risk of bias

Describe the reference standard and how it was conducted and interpreted:
Any reported death was considerd to be a reference standard

- Is the reference standard likely to correctly classify the target condition? Yes
- Were the reference standard results interpreted without knowledge of the results of the index test? Yes

Could the reference standard, its conduct, or its interpretation have introduced bias? **RISK: LOW**

a. Concerns regarding applicability

Is there concern that the target condition as defined by the reference standard does not match the review question? **CONCERN: LOW**

Domain 4: Flow and timing

a. Risk of bias

Describe any patients who did not receive the index test(s) and/or reference standard or who were excluded from the 2x2 table (refer to flow diagram):<br>*No excluded patients reported*<br><br>*Describe the time interval and any interventions between index test(s) and reference standard:*<br>*NR*

• Was there an appropriate interval between index test(s) and reference standard?	Yes
• Did all patients receive a reference standard?	Yes
• Did patients receive the same reference standard?	Yes
• Were all patients included in the analysis?	Yes

Could the patient flow have introduced bias? **RISK: LOW**

Domain 1: Patient selection

A. Risk of bias

Describe methods of patient selection:
Children aged 6 months-15 years. Inclusion criteria: P. falciparum in the thick blood film and 1≥ of following conditions: prostration, coma, hypoglycemia, repeated generalized convulsions, pulmonary edema/respiratory distress, spontanous bleeding, renal failure, severe anemia

- Was a consecutive or random sample of patients enrolled? Yes
- Was a case-control design avoided? Yes
- Did the study avoid inappropriate exclusions? Yes

Could the selection of patients have introduced bias? RISK: LOW

B. Concerns regarding applicability

Describe included patients (prior testing, presentation, intended use of index test and setting):
See above

Is there concern that the included patients do not match the review question? CONCERN: LOW

Domain 2a: Clinical index tests

A. Risk of bias

Describe the index test and how it was conducted and interpreted:
Convulsions: >2 in last 24h, coma (BCS<2). No details on respiratory distress/pulmonary edema

- Were the index test results interpreted without knowledge of the results of the reference standard? Yes
- Were the index test results collected prospectively? Yes
- Were the index test results established and reported using standarized clinical procedures and data collection tools Unclear

Could the conduct or interpretation of the index test have introduced bias? RISK:UNCLEAR

B. Concerns regarding applicability

Is there concern that the index test, its conduct, or interpretation differ from the review question? CONCERN:UNCLEAR

Domain 2b: Laboratory index tests

A. Risk of bias

Describe the index test and how it was conducted and interpreted:
Blood sample drawn on admission

- Were the index test results interpreted without knowledge of the results of the reference standard? — Yes
- Were the index test results collected prospectively? — Yes
- Were the index test results established and reported using standarized clinical procedures and data collection tools? — Yes
- If a treshold was used was it pre-specified? — Yes

Could the conduct or interpretation of the index test have introduced bias? **RISK:LOW**

B. Concerns regarding applicability

Is there concern that the index test, its conduct, or interpretation differ from the review question? **CONCERN: LOW**

Domain 3: Reference standard

A. Risk of bias

Describe the reference standard and how it was conducted and interpreted:
Any reported death was considerd to be a reference standard

- Is the reference standard likely to correctly classify the target condition? — Yes
- Were the reference standard results interpreted without knowledge of the results of the index test? — Yes

Could the reference standard, its conduct, or its interpretation have introduced bias? **RISK: LOW**

B. Concerns regarding applicability

Is there concern that the target condition as defined by the reference standard does not match the review question? **CONCERN: LOW**

Domain 4: Flow and timing

A. Risk of bias

Describe any patients who did not receive the index test(s) and/or reference standard or who were excluded from the 2x2 table (refer to flow diagram):<br>*Excluded: patients with unkown outcome (14%)*<br><br>*Describe the time interval and any interventions between index test(s) and reference standard:*<br>*NR*

• Was there an appropriate interval between index test(s) and reference standard?	Yes
• Did all patients receive a reference standard?	Yes
• Did patients receive the same reference standard?	Yes
• Were all patients included in the analysis?	Yes
**Could the patient flow have introduced bias?**	**RISK: LOW**

QUADAS-2 tool: Risk of bias and applicability judgments   Study reference: VARANDAS 2000

Domain 1: Patient selection

A. Risk of bias

Describe methods of patient selection:

Children aged 6-72 months with cerebral malaria and other forms of sevre malaria based on WHO definition (1990) and confirmed by parasitemia

- Was a consecutive or random sample of patients enrolled? Yes
- Was a case-control design avoided? Yes
- Did the study avoid inappropriate exclusions? No

Could the selection of patients have introduced bias? **RISK: HIGH**

B. Concerns regarding applicability

Describe included patients (prior testing, presentation, intended use of index test and setting):

See above

Is there concern that the included patients do not match the review question? **CONCERN: LOW**

Domain 2: Clinical index tests

A. Risk of bias

Describe the index test and how it was conducted and interpreted:

Conducted on admission, recorded on standarized forms; coma (BCS<4) interpreted as unable to localize painful stimulus; no precise definition regarding number of convulsions

- Were the index test results interpreted without knowledge of the results of the reference standard? Yes
- Were the index test results collected prospectively? Yes
- Were the index test results established and reported using standarized clinical procedures and data collection tools? Yes

Could the conduct or interpretation of the index test have introduced bias? **RISK:LOW**

B. Concerns regarding applicability

Is there concern that the index test, its conduct, or interpretation differ from the review question? **CONCERN:LOW**

Domain 3: Reference standard

A. Risk of bias

Describe the reference standard and how it was conducted and interpreted:
Any reported death was considerd to be a reference standard

- Is the reference standard likely to correctly classify the target condition? Yes
- Were the reference standard results interpreted without knowledge of the results of the index test? Yes

Could the reference standard, its conduct, or its interpretation have introduced bias? **RISK: LOW**

B. Concerns regarding applicability

Is there concern that the target condition as defined by the reference standard does not match the review question? **CONCERN: LOW**

Domain 4: Flow and timing

A. Risk of bias

Describe any patients who did not receive the index test(s) and/or reference standard or who were excluded from the 2x2 table (refer to flow diagram):
Excluded: 6 patients with neurological sequelae by discharge;
Describe the time interval and any interventions between index test(s) and reference standard:
Mean time before death: 20.5 h

- Was there an appropriate interval between index test(s) and reference standard? Yes
- Did all patients receive a reference standard? Yes
- Did patients receive the same reference standard? Yes
- Were all patients included in the analysis? Yes

Could the patient flow have introduced bias? **RISK: LOW**

Domain 1: Patient selection

A. Risk of bias

Describe methods of patient selection:

All children (0-15 years old) with clinical signs of malaria and a P.falciparum-positive thick blood film; presence of WHO severity criteria or respiratory distress documented by a physician on ad hoc designed forms; pediatric department

- Was a consecutive or random sample of patients enrolled? Yes
- Was a case-control design avoided? Yes
- Did the study avoid inappropriate exclusions? Yes

Could the selection of patients have introduced bias? **RISK: LOW**

B. Concerns regarding applicability

Describe included patients (prior testing, presentation, intended use of index test and setting):

See above

Is there concern that the included patients do not match the review question? **CONCERN: LOW**

Domain 2: Laboratory index test

A. Risk of bias

Describe the index test and how it was conducted and interpreted:

Blood sample drawn on admission, definition: platelet count< 100 000/mm3

- Were the index test results interpreted without knowledge of the results of the reference standard? Yes
- Were the index test results collected prospectively? Yes
- Were the index test results established and reported using standarized clinical procedures and data collection tools? Yes
- If a treshold was used was it pre-specified? Yes

Could the conduct or interpretation of the index test have Introduced bias? **RISK: LOW**

B. Concerns regarding applicability

Is there concern that the index test, its conduct, or interpretation differ from the review question? **CONCERN:LOW**

Domain 3: Reference standard

A. Risk of bias

Describe the reference standard and how it was conducted and interpreted:
Any reported death was considerd to be a reference standard

- Is the reference standard likely to correctly classify the target condition? Yes
- Were the reference standard results interpreted without knowledge of the results of the index test? Yes

Could the reference standard, its conduct, or its interpretation have introduced bias? **RISK: LOW**

B. Concerns regarding applicability

Is there concern that the target condition as defined by the reference standard does not match the review question? **CONCERN: LOW**

Domain 4: Flow and timing

A. Risk of bias

Describe any patients who did not receive the index test(s) and/or reference standard or who were excluded from the 2x2 table (refer to flow diagram):<br>*None*
Describe the time interval and any interventions between index test(s) and reference standard:<br>*NR*

- Was there an appropriate interval between index test(s) and reference standard? Yes
- Did all patients receive a reference standard? Yes
- Did patients receive the same reference standard? Yes
- Were all patients included in the analysis? Yes

Could the patient flow have introduced bias? **RISK: LOW**

QUADAS-2 tool: Risk of bias and applicability judgments **Study reference: IMBERT 2003**

Domain 1: Patient selection

A. Risk of bias

Describe methods of patient selection:

All patients admitted to pediatric department with severe malaria (based on 1990 and 2000 WHO definition);

- Was a consecutive or random sample of patients enrolled? Yes
- Was a case-control design avoided? Yes
- Did the study avoid inappropriate exclusions? Yes

Could the selection of patients have introduced bias? RISK: LOW

B. Concerns regarding applicability

Describe included patients (prior testing, presentation, intended use of index test and setting):

See above

Is there concern that the included patients do not match the review question? CONCERN: LOW

Domain 2a: Clinical index tests

A. Risk of bias

Describe the index test and how it was conducted and interpreted:

Conducted during the fisrt 24 h of hospitalisation, level of consciousness evaluated using BCS or GCS, WHO severe malaria or respiratory distress resported systematically by one physician

- Were the index test results interpreted without knowledge of the results of the reference standard? Yes
- Were the index test results collected prospectively? Yes
- Were the index test results established and reported using standarized clinical procedures and data collection tools? Yes

Could the conduct or interpretation of the index test have introduced bias? RISK:LOW

B.Concerns regarding applicability

Is there concern that the index test, its conduct, or interpretation differ from the review question? CONCERN:LOW

Domain 2b: Laboratory index tests

A. Risk of bias

Describe the index test and how it was conducted and interpreted:<br>*Blood sample drawn within the first 24 h of hospitalisation*

- Were the index test results interpreted without knowledge of the results of the reference standard? Yes
- Were the index test results collected prospectively? Yes
- Were the index test results established and reported using standarized clinical procedures and data collection tools? Yes
- If a treshold was used was it pre-specified? Yes

Could the conduct or interpretation of the index test have introduced bias? **RISK:LOW**

B. Concerns regarding applicability

Is there concern that the index test, its conduct, or interpretation differ from the review question? **CONCERN: LOW**

Domain 3: Reference standard

A. Risk of bias

Describe the reference standard and how it was conducted and interpreted:<br>*Any reported death was considerd to be a reference standard*

- Is the reference standard likely to correctly classify the target condition? Yes
- Were the reference standard results interpreted without knowledge of the results of the index test? Yes

Could the reference standard, its conduct, or its interpretation have introduced bias? **RISK: LOW**

B. Concerns regarding applicability

Is there concern that the target condition as defined by the reference standard does not match the review question? **CONCERN: LOW**

Domain 4: Flow and timing

A. Risk of bias

Describe any patients who did not receive the index test(s) and/or reference standard or who were excluded from the 2x2 table (refer to flow diagram):<br>*286/316 (91%) patients tested for thrombocytopenia, otherwise no further exclusions*<br><br>*Describe the time interval and any interventions between index test(s) and reference standard:*<br>*NR*

• Was there an appropriate interval between index test(s) and reference standard?	Yes
• Did all patients receive a reference standard?	Yes
• Did patients receive the same reference standard?	Yes
• Were all patients included in the analysis?	Yes
**Could the patient flow have introduced bias?**	**RISK: LOW**

Domain 1: Patient selection

A. Risk of bias

Describe methods of patient selection:

children admitted to high-dependency unit by medically qualified memebers, who completed standard admission questionnaire and examination

- Was a consecutive or random sample of patients enrolled? Yes
- Was a case-control design avoided? Yes
- Did the study avoid inappropriate exclusions? Yes

Could the selection of patients have introduced bias? **RISK: LOW**

B. Concerns regarding applicability

Describe included patients (prior testing, presentation, intended use of index test and setting):

Patients with blood-film-positive P.falciparum with 1≥ of following features: prostration, coma, prolonged or recurrent seizures, respiratory distress, circulatory collapse, anemia, jaundice

Is there concern that the included patients do not match the review question? **CONCERN: LOW**

Domain 2a: Clinical index tests

A. Risk of bias

Describe the index test and how it was conducted and interpreted:

prostration inability to sit or breast feed, impaired consciousness: prostration or BCS≤2, circulatory collapse: shock score ≥2

- Were the index test results interpreted without knowledge of the results of the reference standard? Yes
- Were the index test results collected prospectively? Yes
- Were the index test results established and reported using standarized clinical procedures and data collection tools? Yes

Could the conduct or interpretation of the index test have introduced bias? **RISK:LOW**

B. Concerns regarding applicability

Is there concern that the index test, its conduct, or interpretation differ from the review question? **CONCERN: LOW**

Domain 2b: Laboratory index tests: anemia, hypoglycemia, renal failure

A. Risk of bias

Describe the index test and how it was conducted and interpreted:
Acidosis: BE <-8 and/or deep breathing, anemia: hemoglobin<5 g/dL

• Were the index test results interpreted without knowledge of the results of the reference standard?	Yes
• Were the index test results collected prospectively?	Yes
• Were the index test results established and reported using standarized clinical procedures and data collection tools?	Yes
• If a treshold was used was it pre-specified?	Yes

Could the conduct or interpretation of the index test have introduced bias? **RISK:LOW**

B. Concerns regarding applicability

Is there concern that the index test, its conduct, or interpretation differ from the review question? **CONCERN: UNCLEAR**

Domain 3: Reference standard

A. Risk of bias

Describe the reference standard and how it was conducted and interpreted:
Any reported death was considerd to be a reference standard

• Is the reference standard likely to correctly classify the target condition?	Yes
• Were the reference standard results interpreted without knowledge of the results of the index test?	Yes

Could the reference standard, its conduct, or its interpretation have introduced bias? **RISK: LOW**

B. Concerns regarding applicability

Is there concern that the target condition as defined by the reference standard does not match the review question? **CONCERN: LOW**

Domain 4: Flow and timing

A. Risk of bias

Describe any patients who did not receive the index test(s) and/or reference standard or who were excluded from the 2x2 table (refer to flow diagram):<br>*No excluded patients*<br><br>*Describe the time interval and any interventions between index test(s) and reference standard:*<br>*NR*

• Was there an appropriate interval between index test(s) and reference standard?	Yes
• Did all patients receive a reference standard?	Yes
• Did patients receive the same reference standard?	Yes
• Were all patients included in the analysis?	Yes
**Could the patient flow have introduced bias?**	**RISK: LOW**

QUADAS-2 tool: Risk of bias and applicability judgments   Study ref: MOCKENHAUPT 2004

Domain 1: Patient selection

A. Risk of bias

Describe methods of patient selection:
Children aged 6 months to 9 years with signs of severe malaria were examined by one of the authors

- Was a consecutive or random sample of patients enrolled? Yes
- Was a case-control design avoided? Yes
- Did the study avoid inappropriate exclusions? Yes

Could the selection of patients have introduced bias? **RISK: LOW**

B. Concerns regarding applicability

Describe included patients (prior testing, presentation, intended use of index test and setting):
asexual P.falciparum parasitemia, and ≤1 of the following WHO (2000) criteria: severe anemia, prostration, respiratory distress, multiple convulsions, impaired consciousness, hemoglobinuria, clinical jaundice, circulatory collapse, abnormal bleeding, pulmonary edema

Is there concern that the included patients do not match the review question? **CONCERN: LOW**

Domain 2a: Clinical index tests

A. Risk of bias

Describe the index test and how it was conducted and interpreted:
prostration: inability to sit or eat, respiratory distress: nasal flaring or Kussmaul breathing or subcostal recession, convulsions: history within preceding 24 h and one directly observed, impaired consciousnes: BSC≤4, hemoglobinuria: dipstick, cicrulatory collapse: SBP<60 mmHg in ≤5 years old children, SBP<80 mmHg in > 5 years old children, hyperpyrexia: >40°C

- Were the index test results interpreted without knowledge of the results of the reference standard? Yes
- Were the index test results collected prospectively? Yes
- Were the index test results established and reported using standarized clinical procedures and data collection tools? Unclear

Could the conduct or interpretation of the index test have introduced bias? **RISK:LOW**

B. Concerns regarding applicability

Is there concern that the index test, its conduct, or interpretation differ from the review question? **CONCERN: LOW**

Domain 2b: Laboratory index tests: anemia, hypoglycemia, renal failure

A. Risk of bias

Describe the index test and how it was conducted and interpreted:
Anema: Hb<5g/dL, hypoglycemia< 2.2mmol/L, hyperlactatemia≥5 mmol/L

• Were the index test results interpreted without knowledge of the results of the reference standard?	Yes
• Were the index test results collected prospectively?	Yes
• Were the index test results established and reported using standarized clinical procedures and data collection tools?	Unclear
• If a treshold was used was it pre-specified?	Yes

Could the conduct or interpretation of the index test have introduced bias? **RISK:LOW**

B. Concerns regarding applicability

Is there concern that the index test, its conduct, or interpretation differ from the review question? **CONCERN: LOW**

Domain 3: Reference standard

A. Risk of bias

Describe the reference standard and how it was conducted and interpreted:
Any reported death was considerd to be a reference standard

• Is the reference standard likely to correctly classify the target condition?	Yes
• Were the reference standard results interpreted without knowledge of the results of the index test?	Yes

Could the reference standard, its conduct, or its interpretation have introduced bias? **RISK: LOW**

B. Concerns regarding applicability

Is there concern that the target condition as defined by the reference standard does not match the review question? **CONCERN: LOW**

Domain 4: Flow and timing

A. Risk of bias

Describe any patients who did not receive the index test(s) and/or reference standard or who were excluded from the 2x2 table (refer to flow diagram):<br>*5 patients dropped out of the study*<br><br>*Describe the time interval and any interventions between index test(s) and reference standard:*<br>*Twenty children died in 24 hours, seven children within 24-48 hours, five children within 48-72 hours following hospitalization*

• Was there an appropriate interval between index test(s) and reference standard?	Yes
• Did all patients receive a reference standard?	Yes
• Did patients receive the same reference standard?	Yes
• Were all patients included in the analysis?	Yes
**Could the patient flow have introduced bias?**	**RISK: LOW**

QUADAS-2 tool: Risk of bias and applicability judgments   Study ref: DZEING-ELLA 2005

Domain 1: Patient selection

A. Risk of bias

Describe methods of patient selection:
Febrile children (or those with history of fever in the last 48 hours), aged 0-10 years of age, >2 asexual forms of P. falciparum on blood film and one or more of the following features: BCS≤2, convulsions, hyperlactatemia, hypoglycemia, severe anaemia; seen on admission by a clinician, summary data recorded on a pro-forma sheet

- Was a consecutive or random sample of patients enrolled? Yes
- Was a case-control design avoided? Yes
- Did the study avoid inappropriate exclusions? No

Could the selection of patients have introduced bias? **RISK: LOW**

B. Concerns regarding applicability

Describe included patients (prior testing, presentation, intended use of index test and setting):
See above

Is there concern that the included patients do not match the review question? **CONCERN: LOW**

Domain 2a: Clinical index tests

A. Risk of bias

Describe the index test and how it was conducted and interpreted:
Cerebral malaria: BCS≤2, convulsions: 3≥ in 24 h, respiratory distress: abnormalities in RR or rhythm or signs of dsitress such as nasal flaring, intercostal or subcostal recession

- Were the index test results interpreted without knowledge of the results of the reference standard? Yes
- Were the index test results collected prospectively? Yes
- Were the index test results established and reported using standarized clinical procedures and data collection tools? Yes

Could the conduct or interpretation of the index test have introduced bias? **RISK:LOW**

B. Concerns regarding applicability

Is there concern that the index test, its conduct, or interpretation differ from the review question? **CONCERN: LOW**

Domain 2b: Laboratory index tests

A. Risk of bias

Describe the index test and how it was conducted and interpreted:
Lactate and glucose measured within 15 minutes of blood sampling; anemia: Hb<5g/dL, hypoglycemia< 2.2mmol/L, hyperlactatemia≥5 mmol/L

• Were the index test results interpreted without knowledge of the results of the reference standard?	Yes
• Were the index test results collected prospectively?	Yes
• Were the index test results established and reported using standarized clinical procedures and data collection tools?	Yes
• If a treshold was used was it pre-specified?	Yes

Could the conduct or interpretation of the index test have introduced bias? **RISK:LOW**

B. Concerns regarding applicability

Is there concern that the index test, its conduct, or interpretation differ from the review question? **CONCERN: LOW**

Domain 3: Reference standard

A. Risk of bias

Describe the reference standard and how it was conducted and interpreted:
Any reported death was considerd to be a reference standard

• Is the reference standard likely to correctly classify the target condition?	Yes
• Were the reference standard results interpreted without knowledge of the results of the index test?	Yes

Could the reference standard, its conduct, or its interpretation have introduced bias? **RISK: LOW**

B. Concerns regarding applicability

Is there concern that the target condition as defined by the reference standard does not match the review question? **CONCERN: LOW**

Domain 4: Flow and timing

A. Risk of bias

Describe any patients who did not receive the index test(s) and/or reference standard or who were excluded from the 2x2 table (refer to flow diagram):<br>*7 children lost to follow-up, 463/576 (80%) of children with available blood lactate measure*<br><br>*Describe the time interval and any interventions between index test(s) and reference standard:*<br>*90% of deaths within the first 24 h gollowing admission*

• Was there an appropriate interval between index test(s) and reference standard?	Yes
• Did all patients receive a reference standard?	Yes
• Did patients receive the same reference standard?	Yes
• Were all patients included in the analysis?	Yes
**Could the patient flow have introduced bias?**	**RISK: LOW**

Domain 1: Patient selection

A. Risk of bias

Describe methods of patient selection:
Children aged 3 months to 5 years with suspicion of malaria, admitted to paediatric ward, clinical examination by doctor or medical student in final year

- Was a consecutive or random sample of patients enrolled? Yes
- Was a case-control design avoided? Yes
- Did the study avoid inappropriate exclusions? No

Could the selection of patients have introduced bias? RISK: HIGH

B. Concerns regarding applicability

Describe included patients (prior testing, presentation, intended use of index test and setting):
P.falciparum and at least one of the following clinical or biological criteria;coma, impaired consciousness, repeated convulsions, prostration, respiratory distress, jaundice, metabolic acidosis, sevre anaemia, hyperparasitemia, microscopic haemoglobinuria, renal failure, collapse, abonormal bleeding or pulmonary odema

Is there concern that the included patients do not match the review question? CONCERN: HIGH

Domain 2a: Clinical index tests

A. Risk of bias

Describe the index test and how it was conducted and interpreted:
coma (BCS≤2), impaired consciousness (BCS >2 and <5); no description of other index tests

- Were the index test results interpreted without knowledge of the results of the reference standard? Yes
- Were the index test results collected prospectively? Yes
  Were the index test results established and reported using standarized clinical procedures and data collection tools? Unclear

Could the conduct or interpretation of the index test have introduced bias? RISK:UNCLEAR

B. Concerns regarding applicability

Is there concern that the index test, its conduct, or interpretation differ from the review question? CONCERN: UNCLEAR

Domain 2b: Laboratory index tests

A. Risk of bias

Describe the index test and how it was conducted and interpreted:
Anaemia: Hb<5g/dL, hypoglycemia: blood glucose< 2.2 mmol/L, parasitemi>4 %

• Were the index test results interpreted without knowledge of the results of the reference standard?	Yes
• Were the index test results collected prospectively?	Yes
• Were the index test results established and reported using standarized clinical procedures and data collection tools?	Unclear
• If a treshold was used was it pre-specified?	Yes

Could the conduct or interpretation of the index test have introduced bias? **RISK:LOW**

B. Concerns regarding applicability

Is there concern that the index test, its conduct, or interpretation differ from the review question? **CONCERN: LOW**

Domain 3: Reference standard

B. Risk of bias

Describe the reference standard and how it was conducted and interpreted:
Any reported death was considerd to be a reference standard

• Is the reference standard likely to correctly classify the target condition?	Yes
• Were the reference standard results interpreted without knowledge of the results of the index test?	Yes

Could the reference standard, its conduct, or its interpretation have introduced bias? **RISK: LOW**

C. Concerns regarding applicability

Is there concern that the target condition as defined by the reference standard does not match the review question? **CONCERN: LOW**

Domain 4: Flow and timing

A. Risk of bias

Describe any patients who did not receive the index test(s) and/or reference standard or who were excluded from the 2x2 table (refer to flow diagram):
No excluded patients
Describe the time interval and any interventions between index test(s) and reference standard:
NR

• Was there an appropriate interval between index test(s) and reference standard?	Yes
• Did all patients receive a reference standard?	Yes
• Did patients receive the same reference standard?	Yes
• Were all patients included in the analysis?	Yes
**Could the patient flow have introduced bias?**	**RISK: LOW**

QUADAS-2 tool: Risk of bias and applicability judgments   Study ref: MAITLAND 2005

Domain 1: Patient selection

A. Risk of bias

Describe methods of patient selection:
clinical feature of severe malaria (i.e., prostration, coma, or respiratory distress), and Plasmodium falciparum parasitemia and metabolic acidosis (base deficit >8mmol/L) and Hb>50 g/L; pediatric high-dependency unit

- Was a consecutive or random sample of patients enrolled? Yes
- Was a case-control design avoided? Yes
- Did the study avoid inappropriate exclusions? No

Could the selection of patients have introduced bias? **RISK: HIGH**

B. Concerns regarding applicability

Describe included patients (prior testing, presentation, intended use of index test and setting):
See above

Is there concern that the included patients do not match the review question? **CONCERN: HIGH**

Domain 2a: Laboratory index tests

A. Risk of bias

Describe the index test and how it was conducted and interpreted:
Acidosis: base deficit>15

- Were the index test results interpreted without knowledge of the results of the reference standard? Yes
- Were the index test results collected prospectively? Yes
- Were the index test results established and reported using standarized clinical procedures and data collection tools? Yes
- If a treshold was used was it pre-specified? Yes

Could the conduct or interpretation of the index test have introduced bias? **RISK:LOW**

B. Concerns regarding applicability

Is there concern that the index test, its conduct, or interpretation differ from the review question? **CONCERN: LOW**

Domain 3: Reference standard

A. Risk of bias

Describe the reference standard and how it was conducted and interpreted:

Any reported death was considerd to be a reference standard

- Is the reference standard likely to correctly classify the target condition? — Yes
- Were the reference standard results interpreted without knowledge of the results of the index test? — Yes

Could the reference standard, its conduct, or its interpretation have introduced bias? — **RISK: LOW**

B. Concerns regarding applicability

Is there concern that the target condition as defined by the reference standard does not match the review question? — **CONCERN: LOW**

Domain 4: Flow and timing

A. Risk of bias

Describe any patients who did not receive the index test(s) and/or reference standard or who were excluded from the 2x2 table (refer to flow diagram):

No excluded patients

Describe the time interval and any interventions between index test(s) and reference standard:

NR

- Was there an appropriate interval between index test(s) and reference standard? — Yes
- Did all patients receive a reference standard? — Yes
- Did patients receive the same reference standard? — Yes
- Were all patients included in the analysis? — Yes

Could the patient flow have introduced bias? — **RISK: LOW**

QUADAS-2 tool: Risk of bias and applicability judgments   Study ref: ZEIDAN 2005

Domain 1: Patient selection

A.Risk of bias

Describe methods of patient selection:
Children<15 years old admitted to four hospitals and diagnosed with severe malaria according to WHO criteria; daily records of paediatric admissions and of children with severe malaria reviewed and checked by trained medical officers and verified by a senior paediatrician in each hospital

- Was a consecutive or random sample of patients enrolled? Yes
- Was a case-control design avoided? Yes
- Did the study avoid inappropriate exclusions? Yes

Could the selection of patients have introduced bias? **RISK: LOW**

B.Concerns regarding applicability

Describe included patients (prior testing, presentation, intended use of index test and setting):
See above

Is there concern that the included patients do not match the review question? **CONCERN: LOW**

Domain 2a: Clinical index tests

A. Risk of bias

Describe the index test and how it was conducted and interpreted
hyperpyrexia: ≤40°C, definitions of other index tests not provided, a checklist with clinical information reported for each case by medical officers and checked by senior paediatriacians

- Were the index test results interpreted without knowledge of the results of the reference standard? Yes
- Were the index test results collected prospectively? Yes

  Were the index test results established and reported using standarized clinical procedures and data collection tools? Yes

Could the conduct or interpretation of the index test have introduced bias? **RISK:LOW**

B. Concerns regarding applicability

Is there concern that the index test, its conduct, or interpretation differ from the review question? **CONCERN: LOW**

Domain 2b: Laboratory index tests

A. Risk of bias

Describe the index test and how it was conducted and interpreted:
Leucocytosis: ≥11000/mm3

- Were the index test results interpreted without knowledge of the results of the reference standard? — Yes
- Were the index test results collected prospectively? — Yes
- Were the index test results established and reported using standarized clinical procedures and data collection tools? — Yes
- If a treshold was used was it pre-specified? — Yes

Could the conduct or interpretation of the index test have introduced bias? **RISK:LOW**

B. Concerns regarding applicability

Is there concern that the index test, its conduct, or interpretation **CONCERN: LOW**

Domain 3: Reference standard

A. Risk of bias

Describe the reference standard and how it was conducted and interpreted:
Any reported death was considerd to be a reference standard

- Is the reference standard likely to correctly classify the target condition? — Yes
- Were the reference standard results interpreted without knowledge of the results of the index test? — Yes

Could the reference standard, its conduct, or its interpretation have introduced bias? **RISK: LOW**

B. Concerns regarding applicability

Is there concern that the target condition as defined by the reference standard does not match the review question? **CONCERN: LOW**

Domain 4: Flow and timing

A. Risk of bias

Describe any patients who did not receive the index test(s) and/or reference standard or who were excluded from the 2x2 table (refer to flow diagram):<br>*No excluded patients*<br>*Describe the time interval and any interventions between index test(s) and reference standard:*<br>*NR*

• Was there an appropriate interval between index test(s) and reference standard?	Yes
• Did all patients receive a reference standard?	Yes
• Did patients receive the same reference standard?	Yes
• Were all patients included in the analysis?	Yes
**Could the patient flow have introduced bias?**	**RISK: LOW**

QUADAS-2 tool: Risk of bias and applicability judgments   Study ref: BRONZAN 2007

Domain 1: Patient selection

A.Risk of bias

Describe methods of patient selection:

children ≥6 months with severe malaria during rainy season from 1996 till 2005, paediatric research ward;not all children with severe malaria are admitted to the research ward, research emphasis on cerebral malaria

- Was a consecutive or random sample of patients enrolled? Yes
- Was a case-control design avoided? Yes
- Did the study avoid inappropriate exclusions? No

Could the selection of patients have introduced bias? **RISK: HIGH**

B.Concerns regarding applicability

Describe included patients (prior testing, presentation, intended use of index test and setting):

Final diagnosis of severe malaria based on presence of 1 of 3 syndromes: cerbral malaria, severe malarial anaemia, or CM with SMA

Is there concern that the included patients do not match the review question? **CONCERN: HIGH**

Domain 2a: Clinical index tests

A. Risk of bias

Describe the index test and how it was conducted and interpreted

All children undergo a complete, standarized history and physical examination

Severe anaemia: BCS≥3 and with either 1) a PCV≤10%, 2)a PCV iof 11%-15% with evidence of clinical decompensation, or 3) a PCV of>15% with the requirement of blood transfusion

Cerebral malaria: BCS≤2 persisting for > 2 hours after other identifiable causes of coma have been excluded

- Were the index test results interpreted without knowledge of the results of the reference standard? Yes
- Were the index test results collected prospectively? Yes
  Were the index test results established and reported using standarized clinical procedures and data collection tools? Yes

Could the conduct or interpretation of the index test have introduced bias? **RISK:LOW**

B. Concerns regarding applicability

Is there concern that the index test, its conduct, or interpretation differ from the review question? **CONCERN: LOW**

Domain 2b: Laboratory index tests

A. Risk of bias

Describe the index test and how it was conducted and interpreted:
HIV test

- Were the index test results interpreted without knowledge of the results of the reference standard? Yes
- Were the index test results collected prospectively? Yes
- Were the index test results established and reported using standarized clinical procedures and data collection tools? Yes
- If a treshold was used was it pre-specified? Yes

Could the conduct or interpretation of the index test have introduced bias? **RISK:LOW**

B. Concerns regarding applicability

Is there concern that the index test, its conduct, or interpretation **CONCERN: LOW**

Domain 3: Reference standard

A. Risk of bias

Describe the reference standard and how it was conducted and interpreted:
Any reported death was considerd to be a reference standard

- Is the reference standard likely to correctly classify the target condition? Yes
- Were the reference standard results interpreted without knowledge of the results of the index test? Yes

Could the reference standard, its conduct, or its interpretation have introduced bias? **RISK: LOW**

B. Concerns regarding applicability

Is there concern that the target condition as defined by the reference standard does not match the review question? **CONCERN: LOW**

Domain 4: Flow and timing

A. Risk of bias

Describe any patients who did not receive the index test(s) and/or reference standard or who were excluded from the 2x2 table (refer to flow diagram):
No excluded patients in patients with severe anemia or cerebral malaria 2x2 tables, 1119/1388 (81%) patients with determined HIV status
Describe the time interval and any interventions between index test(s) and reference standard:
NR

• Was there an appropriate interval between index test(s) and reference standard?	Yes
• Did all patients receive a reference standard?	Yes
• Did patients receive the same reference standard?	Yes
• Were all patients included in the analysis?	Yes
**Could the patient flow have introduced bias?**	**RISK: LOW**

QUADAS-2 tool: Risk of bias and applicability judgments   Study ref: ISSIFOU 2007

Domain 1: Patient selection

A.Risk of bias

Describe methods of patient selection:

Children aged 1-120 months with diagnosis "non per os" falciparum malaria (all patients hospitalized for malaria and treated with iv quinine); 2 areas: rural and urban, medical research units

- Was a consecutive or random sample of patients enrolled? Yes
- Was a case-control design avoided? Yes
- Did the study avoid inappropriate exclusions? No

Could the selection of patients have introduced bias? **RISK: HIGH**

B.Concerns regarding applicability

Describe included patients (prior testing, presentation, intended use of index test and setting):

Each inclusion invloved an assessment of various established prognostic features based on 2000 WHO severe malaria definition: coma, convulsions, hypoglycemia, severe anaemia, respiratory distress, prostration, vomiting. All malaria patients divided accordingly into moderate malaria group (BCS=3-4) and severe malaria group (BCS≤2)

Is there concern that the included patients do not match the review question? **CONCERN:UNCLEAR**

Domain 2a: Clinical index tests

A. Risk of bias

Describe the index test and how it was conducted and interpreted

Cerebral malaria : BCS≤2, respiratory distress: presence of abnormalities in RR, rhythm (kussmaul or Cheyne-Stokes breathing), signs of distress such as nasal flaring, subcostal/intersotal recesssion

- Were the index test results interpreted without knowledge of the results of the reference standard? Yes
- Were the index test results collected prospectively? Yes
- Were the index test results established and reported using standarized clinical procedures and data collection tools? Yes

Could the conduct or interpretation of the index test have introduced bias? **RISK:LOW**

B. Concerns regarding applicability

Is there concern that the index test, its conduct, or interpretation differ from the review question? **CONCERN: LOW**

Domain 2b: Laboratory index tests

A. Risk of bias

Describe the index test and how it was conducted and interpreted:
Severe anaemia: Hb<5g/dL, hypoglycemia: <2.2 mmol/L

• Were the index test results interpreted without knowledge of the results of the reference standard?	Yes
• Were the index test results collected prospectively?	Yes
• Were the index test results established and reported using standarized clinical procedures and data collection tools?	Yes
• If a treshold was used was it pre-specified?	Yes

Could the conduct or interpretation of the index test have introduced bias? **RISK:LOW**

B. Concerns regarding applicability

Is there concern that the index test, its conduct, or interpretation **CONCERN: LOW**

Domain 3: Reference standard

A. Risk of bias

Describe the reference standard and how it was conducted and interpreted:
Any reported death was considerd to be a reference standard

• Is the reference standard likely to correctly classify the target condition?	Yes
• Were the reference standard results interpreted without knowledge of the results of the index test?	Yes

Could the reference standard, its conduct, or its interpretation have introduced bias? **RISK: LOW**

B. Concerns regarding applicability

Is there concern that the target condition as defined by the reference standard does not match the review question? **CONCERN: LOW**

Domain 4: Flow and timing

A. Risk of bias

Describe any patients who did not receive the index test(s) and/or reference standard or who were excluded from the 2x2 table (refer to flow diagram):<br>*No excluded patients*<br>*Describe the time interval and any interventions between index test(s) and reference standard:*<br>*NR*

• Was there an appropriate interval between index test(s) and reference standard?	Yes
• Did all patients receive a reference standard?	Yes
• Did patients receive the same reference standard?	Yes
• Were all patients included in the analysis?	Yes
**Could the patient flow have introduced bias?**	**RISK: LOW**

QUADAS-2 tool: Risk of bias and applicability judgments   Study ref: ODURO 2007

Domain 1: Patient selection

A.Risk of bias

Describe methods of patient selection:<br>*All children between 6 and 59 months of age with diagnosis suggestive of acute disease*

- Was a consecutive or random sample of patients enrolled? Yes
- Was a case-control design avoided? Yes
- Did the study avoid inappropriate exclusions? No

Could the selection of patients have introduced bias? RISK: HIGH

B.Concerns regarding applicability

Describe included patients (prior testing, presentation, intended use of index test and setting):<br>*Criteria for diagnosis and enrolment included the standard WHO definition*

Is there concern that the included patients do not match the review question? CONCERN: LOW

Domain 2a: Clinical index tests

A. Risk of bias

Describe the index test and how it was conducted and interpreted:<br>*Coma score : BCS<3, no further detais provided*

- Were the index test results interpreted without knowledge of the results of the reference standard? Yes
- Were the index test results collected prospectively? Yes

  Were the index test results established and reported using standarized clinical procedures and data collection tools? Yes

Could the conduct or interpretation of the index test have introduced bias? RISK:LOW

B. Concerns regarding applicability

Is there concern that the index test, its conduct, or interpretation differ from the review question? CONCERN: UNCLEAR

Domain 2b: Laboratory index tests

A. Risk of bias

Describe the index test and how it was conducted and interpreted:
On admission blood lactate done for all participants. No further details, including treshold, provided

- Were the index test results interpreted without knowledge of the results of the reference standard? Yes
- Were the index test results collected prospectively? Yes
- Were the index test results established and reported using standarized clinical procedures and data collection tools? Yes
- If a treshold was used was it pre-specified? Unclear

Could the conduct or interpretation of the index test have introduced bias? **RISK:LOW**

B. Concerns regarding applicability

Is there concern that the index test, its conduct, or interpretation differ from the review question? **CONCERN: UNCLEAR**

Domain 3: Reference standard

A. Risk of bias

Describe the reference standard and how it was conducted and interpreted:
Any reported death was considerd to be a reference standard

- Is the reference standard likely to correctly classify the target condition? Yes
- Were the reference standard results interpreted without knowledge of the results of the index test? Yes

Could the reference standard, its conduct, or its interpretation have introduced bias? **RISK: LOW**

B. Concerns regarding applicability

Is there concern that the target condition as defined by the reference standard does not match the review question? **CONCERN: LOW**

Domain 4: Flow and timing

A. Risk of bias

Describe any patients who did not receive the index test(s) and/or reference standard or who were excluded from the 2x2 table (refer to flow diagram):<br>*No excluded patients*<br>*Describe the time interval and any interventions between index test(s) and reference standard:*<br>*NR*

- Was there an appropriate interval between index test(s) and reference standard? Yes
- Did all patients receive a reference standard? Yes
- Did patients receive the same reference standard? Yes
- Were all patients included in the analysis? Yes

Could the patient flow have introduced bias? **RISK: LOW**

QUADAS-2 tool: Risk of bias and applicability judgments   Study ref: ORIMADEGUN 2007

Domain 1: Patient selection

A.Risk of bias

Describe methods of patient selection:
Microscopy-confirmed malaria cases, retrospective review, files of all children

- Was a consecutive or random sample of patients enrolled? — Yes
- Was a case-control design avoided? — Yes
- Did the study avoid inappropriate exclusions? — No

Could the selection of patients have introduced bias? — **RISK: HIGH**

B.Concerns regarding applicability

Describe included patients (prior testing, presentation, intended use of index test and setting):
Cases of severe malaria defined according to 2010 WHO criteria

Is there concern that the included patients do not match the review question? — **CONCERN: LOW**

Domain 2a: Clinical index tests

A. Risk of bias

Describe the index test and how it was conducted and interpreted:
NR

- Were the index test results interpreted without knowledge of the results of the reference standard? — Yes
- Were the index test results collected prospectively? — No
  Were the index test results established and reported using standarized clinical procedures and data collection tools? — Yes

Could the conduct or interpretation of the index test have introduced bias? — **RISK:UNCLEAR**

B. Concerns regarding applicability

Is there concern that the index test, its conduct, or interpretation differ from the review question? — **CONCERN: LOW**

Domain 2b: Laboratory index tests

A. Risk of bias

Describe the index test and how it was conducted and interpreted: *NR*

- Were the index test results interpreted without knowledge of the results of the reference standard? Yes
- Were the index test results collected prospectively? Yes
- Were the index test results established and reported using standarized clinical procedures and data collection tools? Yes
- If a treshold was used was it pre-specified? Unclear

Could the conduct or interpretation of the index test have introduced bias? **RISK:UNCLEAR**

B. Concerns regarding applicability

Is there concern that the index test, its conduct, or interpretation differ from the review question? **CONCERN: LOW**

Domain 3: Reference standard

A. Risk of bias

Describe the reference standard and how it was conducted and interpreted: *Any reported death was considerd to be a reference standard*

- Is the reference standard likely to correctly classify the target condition? Yes
- Were the reference standard results interpreted without knowledge of the results of the index test? Yes

Could the reference standard, its conduct, or its interpretation have introduced bias? **RISK: LOW**

B. Concerns regarding applicability

Is there concern that the target condition as defined by the reference standard does not match the review question? **CONCERN: LOW**

Domain 4: Flow and timing

A. Risk of bias

Describe any patients who did not receive the index test(s) and/or reference standard or who were excluded from the 2x2 table (refer to flow diagram):
102 patients excluded as unable to be traced
Describe the time interval and any interventions between index test(s) and reference standard:
NR

• Was there an appropriate interval between index test(s) and reference standard?	Yes
• Did all patients receive a reference standard?	Yes
• Did patients receive the same reference standard?	Yes
• Were all patients included in the analysis?	Yes
**Could the patient flow have introduced bias?**	**RISK: LOW**

QUADAS-2 tool: Risk of bias and applicability judgments — Study ref: BASSAT 2008

Domain 1: Patient selection

A.Risk of bias

Describe methods of patient selection:

Childern <15 years admitted to the hospital, retrospective study; children with malaria included any sign of severe disease (PCV<15%, deep coma, prostration, hypoglycemia, convulsions, respiratory distress), inability to take oral medication, or moderate anaemia with a risk of cardio-respiratory decompensation

- Was a consecutive or random sample of patients enrolled? Yes
- Was a case-control design avoided? Yes
- Did the study avoid inappropriate exclusions? Yes

Could the selection of patients have introduced bias? **RISK: LOW**

B.Concerns regarding applicability

Describe included patients (prior testing, presentation, intended use of index test and setting):

A sub-group of patients with severe malaria was differentiated

Is there concern that the included patients do not match the review question? **CONCERN: LOW**

Domain 2a: Clinical index tests

A. Risk of bias

Describe the index test and how it was conducted and interpreted:

Deep coma: BCS≤2, prostration: inability to sit unaided or to look for mother's breast/feed in children who cannot yet sit, convulsions: ≥2 reported episodes in the 24 hours before admission, respiratory distress: deep breathing or indrawing; a standarized admission questionnaire; a physician or experienced medical officer performed a physical exam of the children on admission and filled the questionnaire.

- Were the index test results interpreted without knowledge of the results of the reference standard? Yes
- Were the index test results collected prospectively? Yes
- Were the index test results established and reported using standarized clinical procedures and data collection tools? Yes

Could the conduct or interpretation of the index test have introduced bias? **RISK:LOW**

B. Concerns regarding applicability

Is there concern that the index test, its conduct, or interpretation differ from the review question? **CONCERN: LOW**

Domain 2b: Laboratory index tests

A. Risk of bias

Describe the index test and how it was conducted and interpreted:
Hypoglycemia: <2.2 momol/L, anaemia: PCV<15%; standarizded admission questionnaire, including laboratory data

• Were the index test results interpreted without knowledge of the results of the reference standard?	Yes
• Were the index test results collected prospectively?	No
• Were the index test results established and reported using standarized clinical procedures and data collection tools?	Yes
• If a treshold was used was it pre-specified?	Yes

Could the conduct or interpretation of the index test have introduced bias? **RISK:LOW**

B. Concerns regarding applicability

Is there concern that the index test, its conduct, or interpretation differ from the review question? **CONCERN: LOW**

Domain 3: Reference standard

A. Risk of bias

Describe the reference standard and how it was conducted and interpreted:
Any reported death was considerd to be a reference standard; all cases of malaria death based on the admission questionnaire were reviewed by a paediatrician and reclassified according to the clinical evolution and other co-existing diagnoses

• Is the reference standard likely to correctly classify the target condition?	Yes
• Were the reference standard results interpreted without knowledge of the results of the index test?	Yes

Could the reference standard, its conduct, or its interpretation have introduced bias? **RISK: LOW**

B. Concerns regarding applicability

Is there concern that the target condition as defined by the reference standard does not match the review question? **CONCERN: LOW**

Domain 4: Flow and timing

A. Risk of bias

Describe any patients who did not receive the index test(s) and/or reference standard or who were excluded from the 2x2 table (refer to flow diagram):<br>*Number of excluded patients<20%*<br>*Describe the time interval and any interventions between index test(s) and reference standard:*<br>*25% of patients died on admission day, >50% of patients died within the first 48 h of arriving to hospital*

• Was there an appropriate interval between index test(s) and reference standard?	Yes
• Did all patients receive a reference standard?	Yes
• Did patients receive the same reference standard?	Yes
• Were all patients included in the analysis?	Yes
**Could the patient flow have introduced bias?**	**RISK: LOW**

Domain 1: Patient selection

A.Risk of bias

Describe methods of patient selection:

All children admitted during the malaria transmission season if admitting physician diagnosed a severe febrile illness supsected to be malaria; all children diagnosed with cerebral malaria and/or severe malarial anaemia were included

- Was a consecutive or random sample of patients enrolled? Yes
- Was a case-control design avoided? Yes
- Did the study avoid inappropriate exclusions? No

Could the selection of patients have introduced bias? RISK: HIGH

B.Concerns regarding applicability

Describe included patients (prior testing, presentation, intended use of index test and setting):

P. falciparum malaria diagnosed if a child had a body temperature >38° together with P. falciparum trophozoites , and no suggestion of other diagnoses by history or clinical examination and simple laboratory investigation; index tests applied only to the group of patients with cerebral malaria or/and severe malarial anemia

Is there concern that the included patients do not match the review question? CONCERN: HIGH

Domain 2a: Clinical index tests

A. Risk of bias

Describe the index test and how it was conducted and interpreted:

Cerebral malaria: BCS<3 persisting for ≥30 minutes and/or occurence of ≥2 seizures in last 24 h and no other cause of seizure or coma such; severe malarial anaemia: PCV<15% or Hb<5 g/dL; respiratory distress:alar flaring or chest recession or use of accessory respiraory muscles or abnormally deep breathing; dehydration decreased skin turgor or delayed capillary refill time or sunken eyes or dry mucous membranes or abnsence of tears; spleen and liver: palpation; a sick child evaluated within 15 minutes of referral, medical history/ examination findings recorded on a standarized sheet

- Were the index test results interpreted without knowledge of the results of the reference standard? Yes
- Were the index test results collected prospectively? Yes
- Were the index test results established and reported using standarized clinical procedures and data collection tools? Yes

Could the conduct or interpretation of the index test have introduced bias? RISK:LOW

B. Concerns regarding applicability

Is there concern that the index test, its conduct, or interpretation differ from the review question? CONCERN: LOW

Domain 2b: Laboratory index tests

A. Risk of bias

Describe the index test and how it was conducted and interpreted:
Hypogylcemia: blood glucose<2.6 mmol/L, sever anemia: PCV <15% or Hb <5 g/dL)

- Were the index test results interpreted without knowledge of the results of the reference standard? — Yes
- Were the index test results collected prospectively? — Yes
- Were the index test results established and reported using standarized clinical procedures and data collection tools? — Yes
- If a treshold was used was it pre-specified? — Yes

Could the conduct or interpretation of the index test have introduced bias? **RISK:LOW**

B. Concerns regarding applicability

Is there concern that the index test, its conduct, or interpretation differ from the review question? **CONCERN: LOW**

Domain 3: Reference standard

A. Risk of bias

Describe the reference standard and how it was conducted and interpreted:
Any reported death was considerd to be a reference standard

- Is the reference standard likely to correctly classify the target condition? — Yes
- Were the reference standard results interpreted without knowledge of the results of the index test? — Yes

Could the reference standard, its conduct, or its interpretation have introduced bias? **RISK: LOW**

B. Concerns regarding applicability

Is there concern that the target condition as defined by the reference standard does not match the review question? **CONCERN: LOW**

Domain 4: Flow and timing

A. Risk of bias

Describe any patients who did not receive the index test(s) and/or reference standard or who were excluded from the 2x2 table (refer to flow diagram):
No excluded patients
Describe the time interval and any interventions between index test(s) and reference standard:
50% of children died within 12 hours

• Was there an appropriate interval between index test(s) and reference standard?	Yes
• Did all patients receive a reference standard?	Yes
• Did patients receive the same reference standard?	Yes
• Were all patients included in the analysis?	Yes
**Could the patient flow have introduced bias?**	**RISK: LOW**

QUADAS-2 tool: Risk of bias and applicability judgments — Study ref: OGETII 2010

Domain 1: Patient selection

A.Risk of bias

Describe methods of patient selection:

Retrospective review of case notes of all children with severe malaria, paediatric high dependency unit; case notes of unselected children fulfilling strictly-defined criteria for severe malaria (P.falciparum and impaired consciousness/respiratory distress) were reviewed

- Was a consecutive or random sample of patients enrolled? Yes
- Was a case-control design avoided? Yes
- Did the study avoid inappropriate exclusions? Yes

Could the selection of patients have introduced bias? **RISK: LOW**

B.Concerns regarding applicability

Describe included patients (prior testing, presentation, intended use of index test and setting):

See above

Is there concern that the included patients do not match the review question? **CONCERN: LOW**

Domain 2: Laboratory index tests

A. Risk of bias

Describe the index test and how it was conducted and interpreted:

Hypoglycemia: ≤3 mmol/L, index test received by all paediatric admissions

- Were the index test results interpreted without knowledge of the results of the reference standard? Yes
- Were the index test results collected prospectively? Yes
- Were the index test results established and reported using standarized clinical procedures and data collection tools? Yes
- If a treshold was used was it pre-specified? Yes

Could the conduct or interpretation of the index test have introduced bias? **RISK:LOW**

B. Concerns regarding applicability

Is there concern that the index test, its conduct, or interpretation differ from the review question? **CONCERN: LOW**

Domain 3: Reference standard

A. Risk of bias

Describe the reference standard and how it was conducted and interpreted:

Any reported death was considerd to be a reference standard

• Is the reference standard likely to correctly classify the target condition?	Yes
• Were the reference standard results interpreted without knowledge of the results of the index test?	Yes
**Could the reference standard, its conduct, or its interpretation have introduced bias?**	**RISK: LOW**

B. Concerns regarding applicability

**Is there concern that the target condition as defined by the reference standard does not match the review question?**	**CONCERN: LOW**

Domain 4: Flow and timing

A. Risk of Bias

Describe any patients who did not receive the index test(s) and/or reference standard or who were excluded from the 2x2 table (refer to flow diagram):

No excluded patients

Describe the time interval and any interventions between index test(s) and reference standard:

NR

• Was there an appropriate interval between index test(s) and reference standard?	Yes
• Did all patients receive a reference standard?	Yes
• Did patients receive the same reference standard?	Yes
• Were all patients included in the analysis?	Yes
**Could the patient flow have introduced bias?**	**No**

QUADAS-2 tool: Risk of bias and applicability judgments | Study ref: CAMARA 2011

Domain 1: Patient selection

A.Risk of bias

Describe methods of patient selection:
All children, 0-15 years

- Was a consecutive or random sample of patients enrolled? Yes
- Was a case-control design avoided? Yes
- Did the study avoid inappropriate exclusions? Yes

Could the selection of patients have introduced bias? **RISK: LOW**

B.Concerns regarding applicability

Describe included patients (prior testing, presentation, intended use of index test and setting):
P. falciparum positive patients with 1≥ severe malaria criteria (2000 WHO)

Is there concern that the included patients do not match the review question? **CONCERN: LOW**

Domain 2a: Clinical index tests

A. Risk of bias

Describe the index test and how it was conducted and interpreted:
All severe malaria crietria evaluated within 24 h following admission; coma: BCS≤2, convulsions: ≥2 in last 24 h , pulmonary oedeme (CXR), circulatory collapse: TAS<60mmHg<5 years or TAS<80 mmHg

- Were the index test results interpreted without knowledge of the results of the reference standard? Yes
- Were the index test results collected prospectively? Yes
- Were the index test results established and reported using standarized clinical procedures and data collection tools? Unclear

Could the conduct or interpretation of the index test have introduced bias? **RISK:UNCLEAR**

B. Concerns regarding applicability

Is there concern that the index test, its conduct, or interpretation differ from the review question? **CONCERN: LOW**

Domain 2b: Laboratory index tests

A. Risk of bias

Describe the index test and how it was conducted and interpreted:
Hypoglycemia. <2.2 mmol/L, hypercreatinemia: >70µmol/L, hemoglobin: ≤5g/dl

- Were the index test results interpreted without knowledge of the results of the reference standard? Yes
- Were the index test results collected prospectively? Yes
- Were the index test results established and reported using

standarized clinical procedures and data collection tools? Unclear

- If a treshold was used was it pre-specified? Yes

Could the conduct or interpretation of the index test have introduced bias? **RISK:UNCLEAR**

B. Concerns regarding applicability

Is there concern that the index test, its conduct, or interpretation differ from the review question? **CONCERN: LOW**

Domain 3: Reference standard

A. Risk of bias

Describe the reference standard and how it was conducted and interpreted:
Any reported death was considerd to be a reference standard

- Is the reference standard likely to correctly classify the target condition? Yes
- Were the reference standard results interpreted without knowledge of the results of the index test? Yes

Could the reference standard, its conduct, or its interpretation have introduced bias? **RISK: LOW**

B. Concerns regarding applicability

Is there concern that the target condition as defined by the reference standard does not match the review question? **CONCERN: LOW**

Domain 4: Flow and timing

A. Risk of bias

Describe any patients who did not receive the index test(s) and/or reference standard or who were excluded from the 2x2 table (refer to flow diagram):
No excluded patients
Describe the time interval and any interventions between index test(s) and reference standard:
NR

- Was there an appropriate interval between index test(s) and reference standard? Yes
- Did all patients receive a reference standard? Yes
- Did patients receive the same reference standard? Yes
- Were all patients included in the analysis? Yes

Could the patients flow have introduced bias? **RISK:LOW**

Domain 1: Patient selection

A.Risk of bias

Describe methods of patient selection:

Children (<15 years) and adults (≥15 years) with suspected severe malaria according to modified WHO criteria, confirmed by blood test

- Was a consecutive or random sample of patients enrolled? Yes
- Was a case-control design avoided? Yes
- Did the study avoid inappropriate exclusions? Yes

Could the selection of patients have introduced bias?

B.Concerns regarding applicability

RISK: LOW

Describe included patients (prior testing, presentation, intended use of index test and setting):

See above

Is there concern that the included patients do not match the review question? **CONCERN: LOW**

Domain 2b: Laboratory index tests

A. Risk of bias

Describe the index test and how it was conducted and interpreted:

HIV-infection: HIV antibody test followed by confirmation test

- Were the index test results interpreted without knowledge of the results of the reference standard? Yes
- Were the index test results collected prospectively? Yes
- Were the index test results established and reported using standarized clinical procedures and data collection tools? Yes
- If a treshold was used was it pre-specified? Yes

Could the conduct or interpretation of the index test have introduced bias? **RISK:LOW**

B. Concerns regarding applicability

Is there concern that the index test, its conduct, or interpretation differ from the review question? **CONCERN: LOW**

Domain 3: Reference standard

A. Risk of bias

Describe the reference standard and how it was conducted and interpreted:
Any reported death was considerd to be a reference standard

- Is the reference standard likely to correctly classify the target condition? Yes
- Were the reference standard results interpreted without knowledge of the results of the index test? Yes

Could the reference standard, its conduct, or its interpretation have introduced bias? **RISK: LOW**

B. Concerns regarding applicability

Is there concern that the target condition as defined by the reference standard does not match the review question? **CONCERN: LOW**

Domain 4: Flow and timing

A. Risk of bias

Describe any patients who did not receive the index test(s) and/or reference standard or who were excluded from the 2x2 table (refer to flow diagram):
Number of excluded patients<20%
Describe the time interval and any interventions between index test(s) and reference standard:
NR

- Was there an appropriate interval between index test(s) and reference standard? Yes
- Did all patients receive a reference standard? Yes
- Did patients receive the same reference standard? Yes
- Were all patients included in the analysis? Yes

Could the patients flow have introduced bias? **RISK:LOW**

Domain 1: Patient selection

A.Risk of bias

Describe methods of patient selection:
Children with signs of severe malaria confiremd by RDT

- Was a consecutive or random sample of patients enrolled? Yes
- Was a case-control design avoided? Yes
- Did the study avoid inappropriate exclusions? Yes

Could the selection of patients have introduced bias? **RISK: LOW**

B.Concerns regarding applicability

Describe included patients (prior testing, presentation, intended use of index test and setting):
Modified severe malaria criteria according to WHO

Is there concern that the included patients do not match the review question? **CONCERN: LOW**

Domain 2a: Clinical index tests

A. Risk of bias

Describe the index test and how it was conducted and interpreted:
Coma: BCS<3, respiratory distress: costal indrawing, use of accessory muscles, nasal alar flaring, deep breathing, or severe tachypnoea, shock: compensated or decompensated

- Were the index test results interpreted without knowledge of the results of the reference standard? Yes
- Were the index test results collected prospectively? Yes
- Were the index test results established and reported using standarized clinical procedures and data collection tools? Yes

Could the conduct or interpretation of the index test have introduced bias? **RISK:LOW**

B. Concerns regarding applicability

Is there concern that the index test, its conduct, or interpretation differ from the review question? **CONCERN: LOW**

Domain 2b: Laboratory index tests

A. Risk of bias

Describe the index test and how it was conducted and interpreted:
Severe anaemia: <5 g/dL, hypoglycemia: <3 mmol/L, acidosis<- 8 mmol/L

- Were the index test results interpreted without knowledge of the results of the reference standard? Yes
- Were the index test results collected prospectively? Yes
- Were the index test results established and reported using standarized clinical procedures and data collection tools? Yes

- If a treshold was used was it pre-specified? Yes

Could the conduct or interpretation of the index test have introduced bias? **RISK:LOW**

B. Concerns regarding applicability

Is there concern that the index test, its conduct, or interpretation differ from the review question? **CONCERN: LOW**

Domain 3: Reference standard

A. Risk of bias

Describe the reference standard and how it was conducted and interpreted:
Any reported death was considerd to be a reference standard

- Is the reference standard likely to correctly classify the target condition? Yes
- Were the reference standard results interpreted without knowledge of the results of the index test? Yes

Could the reference standard, its conduct, or its interpretation have introduced bias? **RISK: LOW**

B. Concerns regarding applicability

Is there concern that the target condition as defined by the reference standard does not match the review question? **CONCERN: LOW**

Domain 4: Flow and timing

A. Risk of bias

Describe any patients who did not receive the index test(s) and/or reference standard or who were excluded from the 2x2 table (refer to flow diagram):
No excluded patients
Describe the time interval and any interventions between index test(s) and reference standard:
NR

- Was there an appropriate interval between index test(s) and reference standard? Yes
- Did all patients receive a reference standard? Yes
- Did patients receive the same reference standard? Yes
- Were all patients included in the analysis? Yes

Could the patients flow have introduced bias? **RISK:LOW**

QUADAS-2 tool: Risk of bias and applicability judgments   Study ref: JALLOW 2012

Domain 1: Patient selection

A.Risk of bias

Describe methods of patient selection:

Children aged 4 months-14 years, blood smear positive for P. falciparum and one or more WHO crietria for severe malaria

- Was a consecutive or random sample of patients enrolled? Yes
- Was a case-control design avoided? Yes
- Did the study avoid inappropriate exclusions? Yes

Could the selection of patients have introduced bias? **RISK: LOW**

B.Concerns regarding applicability

Describe included patients (prior testing, presentation, intended use of index test and setting):

See above

Is there concern that the included patients do not match the review question? **CONCERN: LOW**

Domain 2a: Clinical index tests

A. Risk of bias

Describe the index test and how it was conducted and interpreted:

Respiratory distress: indrawing or use of accessory ,uscles or nasal flaring or deep breathing, convulsions: >3 in last 24 h, decomensated shock: SBP <70 mmHg, prostration: inability to sit unaided in children>7 months, jaundince, hyperpyrexia >40°C

- Were the index test results interpreted without knowledge of the results of the reference standard? Yes
- Were the index test results collected prospectively? Yes
- Were the index test results established and reported using standarized clinical procedures and data collection tools? Yes

Could the conduct or interpretation of the index test have introduced bias? **RISK:LOW**

B. Concerns regarding applicability

Is there concern that the index test, its conduct, or interpretation differ from the review question? **CONCERN: LOW**

Domain 2b: Laboratory index tests

A. Risk of bias

Describe the index test and how it was conducted and interpreted:

Severe anaemia: Hb<50 g/L or PCV<15, hypoglycemia: <2.2 mM, acidosis: plasama bicarbonate <15mmol/L, hyperlactatemia: plasma lactate>5mmol/L, hyperparasitemia: ≥ 500 0000 parasites/μL, renal failure: urine output of >12ml/Kg over 24 h)

- Were the index test results interpreted without knowledge of the results of the reference standard? Yes

- Were the index test results collected prospectively? Yes
- Were the index test results established and reported using standarized clinical procedures and data collection tools? Yes
- If a treshold was used was it pre-specified? Yes

Could the conduct or interpretation of the index test have introduced bias? **RISK:LOW**

B. Concerns regarding applicability

Is there concern that the index test, its conduct, or interpretation differ from the review question? **CONCERN: LOW**

Domain 3: Reference standard

A. Risk of bias

Describe the reference standard and how it was conducted and interpreted:
Any reported death was considerd to be a reference standard

- Is the reference standard likely to correctly classify the target condition?
- Were the reference standard results interpreted without knowledge of the results of the index test?

Could the reference standard, its conduct, or its interpretation have introduced bias?

B. Concerns regarding applicability **RISK: LOW**

Is there concern that the target condition as defined by the reference standard does not match the review question? **CONCERN: LOW**

Domain 4: Flow and timing

A. Risk of bias

Describe any patients who did not receive the index test(s) and/or reference standard or who were excluded from the 2x2 table (refer to flow diagram):
Plasma lactate available in 16% of enrolled patients,evaluation of convultions available in 35% enrolled patients, plasma bicarbonate available in 10% of enrolled patients, blood glucose level available in 70%, evaluation of hypotensive shock performed in 16% of enrolled patients
Describe the time interval and any interventions between index test(s) and reference standard:
NR

- Was there an appropriate interval between index test(s) and reference standard? Yes
- Did all patients receive a reference standard? Yes
- Did patients receive the same reference standard? Yes
- Were all patients included in the analysis? No

Could the patients flow have introduced bias? **RISK: HIGH**

QUADAS-2 tool: Risk of bias and applicability judgments Study ref: VON SEIDLEIN 2012

Domain 1: Patient selection

A.Risk of bias

Describe methods of patient selection:

Children <15 years old with a positive RDT for P.falciparum lactate dehydrogenase with clinically stated severe malaria

- Was a consecutive or random sample of patients enrolled? Yes
- Was a case-control design avoided? Yes
- Did the study avoid inappropriate exclusions? Yes

Could the selection of patients have introduced bias?

B.Concerns regarding applicability

RISK: LOW

Describe included patients (prior testing, presentation, intended use of index test and setting):

See above

Is there concern that the included patients do not match the review question? **CONCERN: LOW**

Domain 2: Clinical index tests

A. Risk of bias

Describe the index test and how it was conducted and interpreted:

Convulsions: 30 min or longer or ≥2 in 24; coma: BCS≤2; prostration: unable to sit unsupported, if<6 months unable to breastfeed; respiratory distress: costal indrawing, use of accessory muscles, nasal alar flaring, deep breathing:labored breathing with abnormally deep chest excursions; shock: compensated or decompensated; chronic disease: lymphadenopathy, malnutrition, candidiasis, severe visible wasting or desquamation

- Were the index test results interpreted without knowledge of the results of the reference standard? Yes
- Were the index test results collected prospectively? Yes
- Were the index test results established and reported using standarized clinical procedures and data collection tools? Yes

Could the conduct or interpretation of the index test have introduced bias? **RISK:LOW**

B. Concerns regarding applicability

Is there concern that the index test, its conduct, or interpretation differ from the review question? **CONCERN: LOW**

Domain 3: Reference standard

A. Risk of bias

Describe the reference standard and how it was conducted and interpreted:
Any reported death was considerd to be a reference standard

- Is the reference standard likely to correctly classify the target condition?
- Were the reference standard results interpreted without knowledge of the results of the index test?

Could the reference standard, its conduct, or its interpretation have introduced bias? **RISK: LOW**

B. Concerns regarding applicability

Is there concern that the target condition as defined by the reference standard does not match the review question? **CONCERN: LOW**

Domain 4: Flow and timing

A. Risk of bias

Describe any patients who did not receive the index test(s) and/or reference standard or who were excluded from the 2x2 table (refer to flow diagram):
Excluded patients<20%
Describe the time interval and any interventions between index test(s) and reference standard:
NR

- Was there an appropriate interval between index test(s) and reference standard? Yes
- Did all patients receive a reference standard? Yes
- Did patients receive the same reference standard? Yes
- Were all patients included in the analysis? Yes

Could the patient flow have introduced bias? **RISK: LOW**

Domain 1: Patient selection

A.Risk of bias

Describe methods of patient selection:

Febrile children (6-59 months) with positive P.falciparum on blood film and features of the WHO case definition for severe malaria

- Was a consecutive or random sample of patients enrolled? Yes
- Was a case-control design avoided? Yes
- Did the study avoid inappropriate exclusions? Yes

Could the selection of patients have introduced bias?

B.Concerns regarding applicability

RISK: LOW

Describe included patients (prior testing, presentation, intended use of index test and setting):

See above

Is there concern that the included patients do not match the review question? **CONCERN: LOW**

Domain 2: Clinical index tests

A. Risk of bias

Describe the index test and how it was conducted and interpreted:

Arterial oxygen saturation measured with an appropriately sized oxygen sensor placed on the right toe or finger; BCS≤2, prostration: inability to sit unspported or the inability to drink or breast-feed in younger children; convulsions (≥2 in last 24 hours, or >30 minutes); respiratory distress (flaring of alar nasi, subcostal or lower chest in-drawing, tachypnea, deep breathing; coca-cola urine; jaundice, hyperpaarasitemia. Data recorded at the time of admission into a structed questionnaire by doctors and research assistants.

- Were the index test results interpreted without knowledge of the results of the reference standard? Yes
- Were the index test results collected prospectively? Yes
- Were the index test results established and reported using standarized clinical procedures and data collection tools? Yes

Could the conduct or interpretation of the index test have introduced bias? **RISK:LOW**

B. Concerns regarding applicability

Is there concern that the index test, its conduct, or interpretation differ from the review question? **CONCERN: LOW**

Domain 2b: Laboratory index tests

C. Risk of bias

Describe the index test and how it was conducted and interpreted:
hypoglycemia (< 3mmol/L); severe anemia (hematocrit <15%); renail failure (urine output <12 ml/kg/24 hours and a serum creatinine >265 µmol/l)

- Were the index test results interpreted without knowledge of the results of the reference standard? Yes
- Were the index test results collected prospectively? Yes
- Were the index test results established and reported using standarized clinical procedures and data collection tools? Yes
- If a treshold was used was it pre-specified? Yes

Could the conduct or interpretation of the index test have introduced bias? **RISK:LOW**

D. Concerns regarding applicability

Is there concern that the index test, its conduct, or interpretation differ from the review question? **CONCERN: LOW**

Domain 3: Reference standard

A. Risk of bias

Describe the reference standard and how it was conducted and interpreted:
Any reported death was considerd to be a reference standard

- Is the reference standard likely to correctly classify the target condition? Yes
- Were the reference standard results interpreted without knowledge of the results of the index test? Yes

Could the reference standard, its conduct, or its interpretation have introduced bias?

B. Concerns regarding applicability **RISK: LOW**

Is there concern that the target condition as defined by the reference standard does not match the review question? **CONCERN: LOW**

Domain 4: Flow and timing

A. Risk of bias

Describe any patients who did not receive the index test(s) and/or reference standard or who were excluded from the 2x2 table (refer to flow diagram):

NR

Describe the time interval and any interventions between index test(s) and reference standard:

NR

• Was there an appropriate interval between index test(s) and reference standrad?	Yes
• Did all patients receive a reference standard?	Yes
• Did patients receive the same reference standard?	Yes
• Were all patients included in the analysis?	Yes

Could the patient flow have introduced bias? **RISK: LOW**

QUADAS-2 tool: Risk of bias and applicability judgments   Study ref: KENDJO 2013

Domain 1: Patient selection

A.Risk of bias

Describe methods of patient selection:
Children with P.falciparum malaria

- Was a consecutive or random sample of patients enrolled?
- Was a case-control design avoided? Yes
- Did the study avoid inappropriate exclusions? Yes

Could the selection of patients have introduced bias? Yes

B.Concerns regarding applicability **RISK: LOW**

Describe included patients (prior testing, presentation, intended use of index test and setting):
See above

Is there concern that the included patients do not match the review question? **CONCERN: HIGH**

Domain 2a: Clinical index tests

A. Risk of bias

Describe the index test and how it was conducted and interpreted:
Coma: BCS≤2; prostration: unable to breastfeed or to sit or stand up or to walk, depending on age

- Were the index test results interpreted without knowledge of the results of the reference standard? Yes
- Were the index test results collected prospectively? Yes
- Were the index test results established and reported using standarized clinical procedures and data collection tools? Yes

Could the conduct or interpretation of the index test have introduced bias? **RISK:LOW**

B. Concerns regarding applicability

Is there concern that the index test, its conduct, or interpretation differ from the review question? **CONCERN: LOW**

Domain 2b: Laboratory index tests

A. Risk of bias

Describe the index test and how it was conducted and interpreted:
Severe anaemia: Hg 5g/L or hematocrit<15%; hypoglycemia: blood glucose>2.2 mmol/L

- Were the index test results interpreted without knowledge of the results of the reference standard? Yes
- Were the index test results collected prospectively? Yes
- Were the index test results established and reported using standarized clinical procedures and data collection tools? Yes

- If a treshold was used was it pre-specified? Yes

Could the conduct or interpretation of the index test have introduced bias? **RISK:LOW**

B. Concerns regarding applicability

Is there concern that the index test, its conduct, or interpretation differ from the review question? **CONCERN: LOW**

Domain 3: Reference standard

A. Risk of bias

Describe the reference standard and how it was conducted and interpreted:<br>*Any reported death was considerd to be a reference standard*

- Is the reference standard likely to correctly classify the target condition? Yes
- Were the reference standard results interpreted without knowledge of the results of the index test? Yes

Could the reference standard, its conduct, or its interpretation have introduced bias? **RISK: LOW**

B. Concerns regarding applicability

Is there concern that the target condition as defined by the reference standard does not match the review question? **CONCERN: LOW**

Domain 4: Flow and timing

Describe any patients who did not receive the index test(s) and/or reference standard or who were excluded from the 2x2 table (refer to flow diagram):<br>*32% of patients without lactate measurement*<br>*Describe the time interval and any interventions between index test(s) and reference standard:*<br>*19 h median time to death*

- **Risk of bias**

- Was there an appropriate interval between index test(s) and reference standard? Yes
- Did all patients receive a reference standard? Yes
- Did patients receive the same reference standard? Yes
- Were all patients included in the analysis? No

Could the patients flow have introduced bias? **RISK: HIGH**
